# Mutualisms within light microhabitats are associated with sensory convergence in a mimetic butterfly community

**DOI:** 10.1073/pnas.2422397122

**Published:** 2025-07-15

**Authors:** J. Benito Wainwright, Theodora Loupasaki, Francisco Ramírez, Iestyn L. Penry-Williams, Sam J. England, Annalie Barker, Joana I. Meier, Martin J. How, Nicholas W. Roberts, Jolyon Troscianko, Stephen H. Montgomery

**Affiliations:** ^a^School of Biological Sciences, Faculty of Life Sciences, University of Bristol, Bristol BS8 1TQ, United Kingdom; ^b^Museo de Zoología Quito Católica Zoología, Laboratorio de Entomología, Escuela de Ciencias Biológicas, Pontificia Universidad Católica del Ecuador, Quito, Ecuador, 170525; ^c^Department of Evolutionary Morphology, Museum für Naturkunde-Leibniz Institute for Evolution and Biodiversity Science, Berlin 10115, Germany; ^d^Department of Zoology, University of Cambridge, Cambridge CB2 3EJ, United Kingdom; ^e^Tree of Life Programme, Wellcome Sanger Institute, Hinxton CB10 1SA, United Kingdom; ^f^Centre for Ecology & Evolution, University of Exeter, Penryn TR10 9EZ, United Kingdom

**Keywords:** convergent evolution, mimicry, niche partitioning, sensory ecology, visual system

## Abstract

Niche partitioning within variable habitats can expose species to distinct sensory information. Vision is the primary sensory modality used by many animals to interact with their habitat. However, the role of terrestrial light environment properties in shaping species assemblages and visual system evolution is not fully understood. By studying a diverse, sympatric community of mimetic butterflies, we demonstrate that forest architecture creates a mosaic of light microhabitats that drive adaptive sensory convergence and divergence in both peripheral and central sensory systems. Our results show that across the visual system, predictable patterns of evolution characterize how species adapt to the availability of sensory information within their preferred light microhabitat. Our study provides an example of how visual systems consistently respond to their external sensory world, and illustrates the wide-reaching consequences of interspecific mutualisms, such as Müllerian mimicry, on organismal evolution.

## Significance

The perception and use of visual information to guide behavior is critical for many animals. Our study establishes how subtle differences in the light environments within tropical forests can drive visual system evolution. We show that ithomiine butterflies that belong to the same mimicry ring also converge on preferences for distinct light microhabitats, and striking similarities in their eye and brain morphology.

Environmental variability is associated with increased biodiversity via adaptive partitioning across multiple ecological dimensions (1). This partitioning exposes species to contrasting ecological challenges, including different light environments (2), which can impact the efficacy of behaviors such as foraging, mating, and predator avoidance (3). In aquatic ecosystems, variation in the light environment, such as those caused by spectral or intensity depth gradients, has been inferred to promote ecological diversification with consequences for adaptive visual system evolution (4–6). While environmental variation in light abundance is also known to exist between terrestrial ecosystems such as highly diverse tropical rainforests (2, 7–11), its sensory complexity and adaptive influence—when modeled through the visual systems of relevant species present in those habitats—remains incompletely characterized. As such, the universal role of ecologically relevant light variation in shaping the adaptive evolution of community structures is largely unknown.

Evolutionary radiations of Neotropical butterflies provide a powerful system for understanding how the light environment drives both niche partitioning and sensory system evolution in terrestrial ecosystems (12). For example, environmental light conditions have been shown to be associated with variation in wing pattern and reflectance (13), the strength of mating preference (14) and investment in sensory structures (15–18) between closely related species. The 26-million-y-old adaptive radiation of the tribe Ithomiini (~400 species, Nymphalidae: Danainae), provides particular promise (19). These species display a striking diversity of morphologies, matched by variation in ecological preferences for host plants and habitat types (20–22). All species are chemically defended and use bright wing coloration to advertise their distastefulness (23). This wing pattern variation can be grouped into discrete categories, reflecting species belonging to the same “mimicry ring,” a group of polyphyletic species with the same warning signal (20, 24). Mimicry rings evolve where unpalatable species converge in wing color pattern, and often also wing morphology and flight behavior, to increase the efficiency of their warning signal to predators (24–27), a phenomenon known as Müllerian mimicry (23). Müllerian mimicry is viewed as a classic example of interspecific mutualism (23, 28, 29), because defended species with similar color patterns benefit by sharing the cost of predator education, thus reducing their individual probability of attack (20, 23). In support of this, in tropical butterflies, color pattern in Müllerian mimics is known to be under positive frequency-dependent selection (27), with shifts in these wing patterns widely considered to initiate pre- and postmating isolation (30–32).

The segregation of predators across microhabitats, combined with habitat-dependent variation in the efficacy of different warning signals, allows multiple mimicry rings to coexist within single, sympatric forest communities (20, 21, 33). This is because mimetic convergence is known to drive convergence in microhabitat use, due to spatial variation in frequency-dependent selection regimes, such that species with similar warning cues show more spatial overlap, increasing the mutual benefit of the mimetic trait by educating a common community of predators (20, 21, 33). Previous work in ithomiines has demonstrated a close correspondence between forest structure and the distribution of ithomiine mimicry rings within sympatric communities, at small spatial ranges (20, 21). Given that forest structure affects the local light environment within a microhabitat (2), we have hypothesized that convergence in microhabitat preference within comimetic ithomiines exposes nonmimetic species to contrasting sensory environments (34, 35). By impacting the efficiency of a broad range of behaviors, from reproduction to foraging, these contrasting sensory conditions could provide a major source of secondary selection for adaptive trait evolution.

Indeed, comparisons among a small sample of species representing the major phylogenetic lineages of ithomiines have revealed strong patterns of variation across a range of visual traits, including at the peripheral (compound eye structure), central (brain structure), and molecular (visual pigment coding sequence) levels, suggesting lability in sensory system evolution across sympatric species with relatively subtle differences in habitat preference (34). In addition, studies focused on investment in sensory brain regions within specific ithomiine genera have provided evidence of sensory specialization, putatively linked to a shift in mimicry ring (35). However, as yet, this variation in sensory traits has not been linked directly to differences in species-specific ecologies, or microhabitat preference behavior. Furthermore, an absence of dense phylogenetic sampling limits our power to generalize, or to understand the broad role of interspecific mimetic mutualisms in indirectly shaping the evolution of visual traits, in both the eye and the brain.

Here, by studying a diverse Ecuadorean community of 54 sympatric ithomiine butterfly species, we address previous limitations by asking whether convergence in mimetic coloration is not only associated with convergence in microhabitat preferences, but that these microhabitats expose species to predictable differences in light regimes, which in turn shapes patterns of visual system evolution. This sympatric community consists of eight mimicry rings, which have been previously shown to segregate across forest microhabitats defined by variation in vegetation (20, 21, 24, 33). We first characterized how heterogeneity in these microhabitats creates variation in local light conditions, and then asked how adaptive species assemblages were strengthened and maintained by abiotic (e.g., changes in the spectral properties of the light environment) and biotic (e.g., mutualistic Müllerian mimicry) interactions. Next, by taking a comparative physiological and anatomical approach, we test the hypothesis that shifts in ambient light preference are associated with adaptive changes in visual system architecture. Our data provide the broad phylogenetic basis, and integrative ecological and sensory data needed to advance our understanding of the impact of variation across light microhabitats on sensory evolution. Our results provide evidence that mutualistic ecological interactions among species that occupy a mosaic patchwork of sensory conditions can drive predictable patterns of visual system evolution.

## Results and Discussion

### Variation in Ecologically Relevant Forest Light Creates a Mosaic of Microhabitats.

**Fig. 1.**
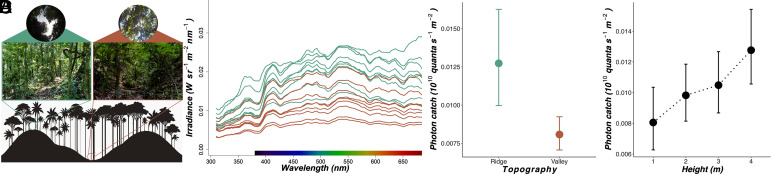
An overview of light environmental differences in tropical rainforests (*N* = 762, 9 ridge/valley replicates). (*A*) Illustration of topographic variation (9 ridges/valleys) along the 2.1 km transect at the field site in Ecuador (*Bottom*), with illustrative digital photographic images from a ridge and valley (*Middle*) and hemispherical, upward facing, 180° fisheye photographs from each location (*Top*). (*B*) Mean calibrated spectral irradiance values within a 310 to 670 nm spectral range for each ridge (green) and valley (brown) replicate along the transect. (*C* and *D*) Mean photon catch (10^10^ quanta s^−1 -−2^) for the LW sensitivity function with respect to transect topography (*C*) and height from the ground (m) (*D*). Error bars represent 95% CI.

We collected spectral irradiance measurements across a 2.1 km topographically variable forest transect, consisting of nine consecutive ridges and valleys, in Parque Nacional Yasuní, Ecuador (Fig. 1*A*). Photon catch [defined here as the absorption of light photons captured by the long-wavelength-sensitive photoreceptor (36)], and the relative number of photons captured by the ultraviolet (UV), blue (B), and long-wavelength (LW) photoreceptors (*Methods*) were estimated using the photoreceptor spectral sensitivities of *Danaus plexippus* (Nymphalidae: Danainae), a closely related species with similar photoreceptor physiology to ithomiines, according to available data (34, 37) (*SI Appendix*, Fig. S1; see *SI Appendix*, *SI Methods*). Modeling spectral irradiance in this way provided a conservative estimate of ecologically relevant achromatic and chromatic visual information within and between forest microhabitats (*Methods*). Overall, the architecture of forest ridges created brighter and broader spectrum visual environments than forest valleys (GLMM: photon catch: χ^2^_1_ = 56.890, *P* < 0.001; Relative B catch, χ^2^_1_ = 17.262, *P* < 0.001; Relative LW catch, χ^2^_1_ = 11.098, *P* = 0.001; Fig. 1 *B* and *C* and Dataset S1). Achromatic photon catch also showed a positive correlation with measurement height (GLMM: χ^2^_1_ = 34.067, *P* < 0.001; Fig. 1*D*) and canopy openness (χ^2^_1_ = 11.023, *P* = 0.001). No interactions between topography, height from the ground, or canopy openness were found for any other spectral variable (Dataset S1).

**Fig. 2.**
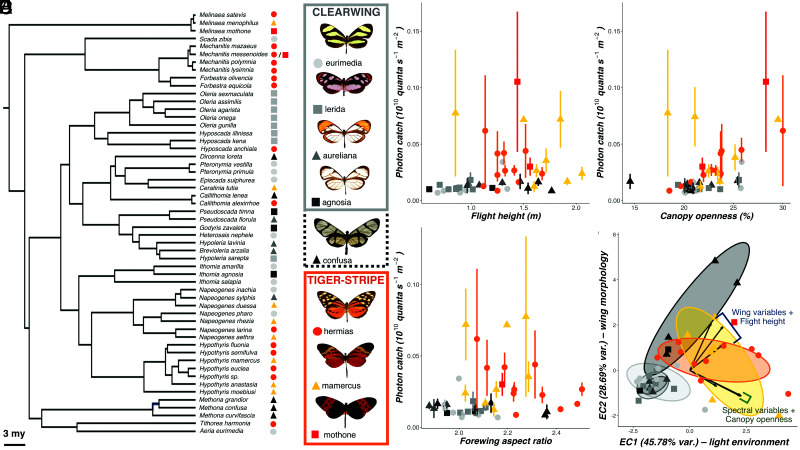
Light microhabitat segregation among ithomiine butterfly mimicry rings. (*A*) A pruned molecular phylogeny (from ref. 19) of the 54 ithomiine species sampled in the Ecuadorean community. Colored symbols at the tips represent the mimicry ring to which each species belongs (light gray circle: “eurimedia,” light gray square: “lerida,” light gray triangle: “aureliana,” dark gray square: “agnosia,” black triangle: “confusa,” orange circle: “hermias,” yellow triangle: “mamercus,” red square: “mothone”). Example models of each mimicry ring are shown on the right, grouped based on their general color pattern classification into the three major color pattern groups (gray: clearwing, orange: tiger-stripe, black: confusa) which are used in later analyses. Confusa has a black dashed border, indicating that it is treated as a separate “mimetic cluster” in later analyses, but it shares the transparent wings of the clearwing group. (*B*–*D*) Mean photon catch (10^10^ quanta s^−1^ m^−2^) for the LW sensitivity function of each species (*N* = 785, 45 species), coded by mimicry ring, plotted against flight height (m) (*B*), canopy openness (%) (*C*), and forewing aspect ratio (*D*). Error bars indicate SE. (*E*) Biplot of ecological axes 1 and 2 (EC1 and EC2) from a principal component analysis (PCA) of mean spectral, ecological, and wing morphological data for each species (*N* = 45 species; *Methods*). Points and ellipses are coded by mimicry ring. Vector lengths are proportional to the variance at that variable and their loadings are summarized using labeled brackets.

To complement our transect data, we recorded spectral irradiance measurements and flight height at the capture location and position of 785 wild, individual ithomiine butterflies (45 species; Fig. 2*A*). This expanded our sampling of the light environment across a broader area (~4.5 km^2^) of forest where butterflies were naturally flying. While controlling for potential phylogenetic effects in species’ visual niches, photon catch, and the relative abundance of blue wavelengths were both positively correlated with canopy openness. We did not see the same effect for flight height, but this is likely explained by aforementioned topographic effects which decouple flight height from distance to the canopy (MCMCglmm: photon catch, *P*-mean = 0.010, 95% CI = 0.007 to 0.013, *P*_MCMC_ < 0.001; Relative B catch, *P*-mean = 0.001, 95% CI = 0.001 to 0.001, *P*_MCMC_ < 0.001; Fig. 2 *B* and *C* and Dataset S2*A*). Additional models using PC scores that separate achromatic and chromatic information largely mirror these results (Datasets S1 and S2*A*). Overall, our results show that the abundance and composition of ecologically important light within tropical forests varies along both vertical (height and topography) and horizontal (canopy openness) axes. Although our light measurements did not account for random noise created by variation in cloud cover, these findings support the broad “forest shade” and “small gap” light microhabitat categories originally assigned by Endler to characterize variation in sunny forest irradiance spectra (2). In summary, open forest microhabitats at higher elevations appear brighter and more spectrally rich to our butterfly visual model compared to closed forest microhabitats at lower elevations.

### Light Microhabitat Partitioning Among Mimicry Rings.

Given prior evidence of ecological convergence, inferred from local variation in vegetation type and height, among mutualistically interacting ithomiine comimics, we next sought to test whether this is true for the axes of light variation we identified (20). Although ithomiine mimicry rings differ in topography and height [MCMCglmm; topography (valley vs. ridge), ΔDIC = 7.856; height, ΔDIC = 5.989; *SI Appendix*, Figs. S2 and S3], our spectral irradiance measurements from individual butterflies revealed finer scale microhabitat partitioning between the eight mimicry rings (MCMCglmm: photon catch, ΔDIC = 10.142; Relative LW catch, ΔDIC = 5.053; Fig. 2 *C* and *D*; Dataset S2*A*). The wing patterns of these eight mimicry rings share similarities that have previously allowed them to be clustered based on their degree of wing transparency (“clearwings”), opaque orange and black coloration (“tiger stripe”) and elongated, robust wings (confusa) (16). Pairwise contrasts between all eight mimicry rings revealed that all mimicry rings with tiger-stripe (opaque; hermias mamercus, mothone mimicry rings) color patterns consistently occupied significantly more illuminated light environments than all mimicry rings with transparent (clearwing and confusa; eurimedia, lerida, aureliana, agnosia, and confusa mimicry rings) color patterns (Fig. 2 and *SI Appendix*, Figs. S3 and S4 and Dataset S2*A*). This suggests a fundamental ecological split mirroring major color pattern traits. Species involved in vividly colored tiger-stripe mimicry rings flew higher, and occupied brighter, broader spectrum and more variable light environments compared to species involved in clearwing mimicry rings, which were confined to shaded forest at lower elevations. Together, our results demonstrate convergence in visual niche preferences associated with mutualistic Müllerian mimicry, generating fine-scale niche partitioning of light environments and maintaining adaptive niche assemblages within tropical forests (38). The observed convergence in light microhabitat is most likely driven by the adaptive evolution of antipredator and conspecific signaling under distinct lighting conditions (39). However, microhabitat segregation among avian predators also mirrors the segregation of ithomiine mimicry rings in this community, suggesting that variation in predator encounter rates could also play a role (33).

### Spectral Variation Is Independent of Flight Behavior.

Ithomiines occupying similar mimicry rings are known to converge in flight-related morphologies, forming three mimetic clusters in flight morphospace within rainforest communities (25). In support of this, we found a positive correlation between forewing aspect ratio and light environment (Fig. 2*D* and Dataset S2*B*). Therefore, variation in flight speed and performance could conceivably drive changes in visual processing independently of light microhabitat preference (25, 40, 41). To disentangle these effects, we conducted a PCA on three major light environmental variables (estimated photon catch for the ultraviolet, blue, and long-wavelength photoreceptors), two ecological variables (flight height and canopy openness), and three flight-related morphological measurements (forewing surface area, aspect ratio, and wing loading; see *Methods*). This resulted in two “ecological axes” (ECs) which explained 45.78% and 28.69% of variation, respectively. All spectral variables loaded most positively onto EC1, and all wing morphological variables loaded most positively onto EC2 (Fig. 2*E* and Dataset S3), providing two independent “light environment” (EC1) and “flight related wing morphology” (EC2) axes which were used in subsequent analyses. All tiger-stripe and clearwing mimicry rings segregated from each other along EC1 but the confusa mimicry ring also diverged along the EC2 “wing morphology” axis, despite occupying similar light environments to the other four clearwing mimicry rings, confirming previous findings of divergence in flight-related morphologies in these species (20) (PGLS: EC1, lambda = 0.000, F_7,37_ = 7.686, *P* < 0.001; EC2, lambda = 0.000, F_7,37_ = 3.659, *P* = 0.004; Fig. 2*E* and Dataset S3). In subsequent analyses, the eight original mimicry rings were therefore aggregated into these three mimetic clusters (clearwing, tiger-stripe, and confusa), based on their consistent segregation along these two ecological axes.

### Light Microhabitat Is Associated with the Evolution of Peripheral Light Reception.

**Fig. 3.**
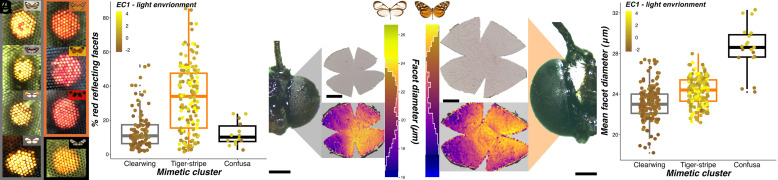
Eye physiological and anatomical associations with light environment and mimicry (*N* = 363, 45 species). (*A*) Example images of the frontal eyeshine from representatives of each mimicry ring within the study community, grouped by mimetic cluster (from left to right: *Ithomia amarilla, Napeogenes larina*, *Oleria gunilla*, *Melinaea menophilus*, *Pseudoscada florula*, *Mechanitis messenoides, Godyris zavaleta*, *Callithomia lenea;* mimetic cluster, gray = clearwing, dotted black = confusa, orange = tiger-stripe). Mimicry ring is indicated in the top right of each panel, with border color denoting mimetic cluster. (*B*) Convergence in the proportion of red reflecting facets (%) for individuals occupying similar light environments, separated by mimetic cluster. (*C*) Example frontal head photographs of *Napeogenes sylphis* (*Left*; mimetic cluster: clearwing) and *Mechanitis mazaeus* (*Right*; mimetic cluster: tiger-stripe) alongside their imaged eye cuticle and its processed output, where individual points denote identified facets, color coded by facet diameter (μm). (Scale bars, 500 µm.) (*D*) Convergence in mean facet diameter for individuals occupying similar light environments, separated by mimetic cluster. For all boxplots, brown-yellow color shades represent mean EC1 values for each species. Medians (thick horizontal bars), interquartile ranges (boxes), values within 1.5 interquartile ranges of the box edges (whiskers), and possible outliers (datapoints outside whiskers) are plotted.

We hypothesized that the patterns we observed in light microhabitat preference would impact the selection regimes acting on the visual system. Using wild-caught butterflies, we first examined physiological data extracted from video recordings of eyeshine, which reveal screening pigments that shift the spectral sensitivity of photoreceptors toward longer wavelengths, resulting in red-reflecting facets (34, 42) (Fig. 3*A*). We found that the relative abundance of red-reflecting facets was positively correlated with EC1, meaning red-reflecting facets were more abundant in species occupying brighter light environments (MCMCglmm: *P*-mean = −0.114, 95% CI = −0.173 to −0.059, P_MCMC_ = 0.001; Fig. 3*B* and *SI Appendix*, Fig. S5 and Dataset S4*A*). Eyeshine exposure is followed by a rapid pupillary response, which we used to assay how rapidly eyes physiologically respond to sudden shifts in lighting conditions. No significant correlation was found between EC1 (or EC2) and the speed of this pupillary response (MCMCglmm: *P*-mean = 0.116, 95% CI = 0.004 to 0.231, *P*_MCMC_ = 0.054), suggesting that differences in temporal light variability between light microhabitats does not explain variation in this trait. However, shifts in screening pigment expression provide a potential evolutionary mechanism, operating in conjunction with the spectral tuning of visual pigments, to shape visual specialization, the physiology of which has been extensively characterized in other butterfly species (34).

We subsequently investigated how these eye physiological adaptations have coevolved with eye structure by measuring the surface area of the eye cuticle, the number of facets, and mean facet diameter (Fig. 3*C*). Eye surface area showed a strong allometric relationship with interocular distance, here used as an allometric control (*SI Appendix*, Figs. S5 and S6; see *SI Appendix*, *SI Methods*), but nevertheless showed a significant nonallometric positive association, independently, with respect to both EC1 (light environment) and EC2 (wing morphology) (MCMCglmm: EC1, *P*-mean = 0.031, 95% CI = 0.019 to 0.043, *P*_MCMC_ < 0.001; EC2, *P*-mean = 0.040, 95% CI = 0.024 to 0.055, *P*_MCMC_ < 0.001; *SI Appendix*, Figs. S5 and S6). Species occupying more illuminated light environments (i.e., species belonging to tiger-stripe mimicry rings) or with forewings equipped for greater flight speeds (i.e., confusa comimics) have convergently evolved larger eyes. When regressing facet number against eye surface area, we again found independent, nonallometric effects of light environment and wing morphology (MCMCglmm: EC1, *P*-mean = 0.008, 95% CI = 0.004 to 0.012, *P*_MCMC_ < 0,001; EC2, *P*-mean = 0.008, 95% CI = 0.003 to 0.014, *P*_MCMC_ = 0.004; *SI Appendix*, Figs. S5 and S6 and Dataset S4*A*), This was confirmed by regressions of mean facet diameter against EC1 and EC2 (MCMCglmm: EC1, *P*-mean = 0.008, 95% CI = 0.004 to 0.011, *P*_MCMC_ < 0.001; EC2, *P*-mean = 0.010, 95% CI = 0.005 to 0.015, *P*_MCMC_ < 0.001; Fig. 3 *C* and *D* and *SI Appendix*, Figs. S5 and S6 and Dataset S4*A*). These results suggest that species with larger eyes have increased both the number of facets and facet diameter, to exploit favorable variation in visual information in well-lit forest. While it remains to be confirmed how these anatomical shifts impact functional performance, the physical structure of the eye has repeatedly evolved in response to increased light abundance found within more open forest microhabitats.

### Light Microhabitat Is Associated with the Evolution of Investment in Visual Brain Centers.

**Fig. 4.**
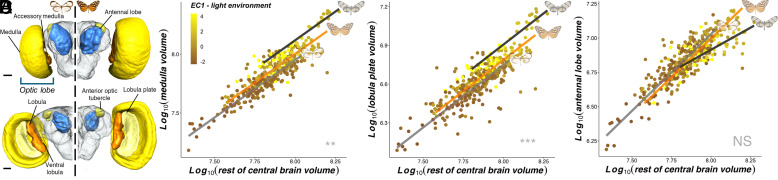
Neuroanatomical associations with light environment and mimicry (*N* = 374 individuals, 40 species). (*A*) Anterior (*Top*) and posterior (*Bottom*) labeled 3D surface reconstructions from the brain of *I. amarilla* (*Left*; mimetic cluster: clearwing) and *M. menophilus* (*Right*; mimetic cluster: tiger-stripe). The figure illustrates all reconstructed neuropils, labeled, and superimposed on an outline of the “rest of central brain.” (Scale bars, 100 µm.) (*B*–*D*) Nonallometric convergence between the mean light environment of each species and the level of volumetric investment (μm^3^) in the medulla (*B*), and lobula plate (*C*), when scaled against the volume of the rest of central brain, with a lack of effect on the antennal lobe (*D*). All lines for each mimetic cluster (gray = clearwing, black = confusa, orange = tiger-stripe), estimated from standardized major axis regressions are superimposed on top. Example models for each mimetic cluster are shown to the right of each line. Asterisks at the bottom right of each panel indicate the significance level of EC1 at predicting relative investment in each neuropil. NS *P* > 0.05, *P < 0.05, **P < 0.01, ***P < 0.001.

Next, we followed the trajectory of visual information to assess whether the light environment specifically impacts the peripheral visual system, or also shapes investment in sensory regions of the central brain (Fig. 4*A*). After accounting for overall brain size (which correlates strongly and positively with other allometric controls; *SI Appendix*, Fig. S7), significant nonallometric associations were found between EC1 and the size of optic lobe neuropils, synapse-dense regions which form the primary visual processing center in the brain (43). In these analyses, investment in the optic lobe as a whole (MCMCglmm: *P*-mean = 0.023, 95% CI = 0.013 to 0.033, *P*_MCMC_ < 0.001), and three of the largest visual neuropils (the medulla, lobula plate, and lobula; Fig. 4 *B* and *C* and *SI Appendix*, Fig. S8 and Dataset S4*A* and S4*B*), were largest in species found in more illuminated microhabitats. An independent, positive, nonallometric effect of EC2 was also found for the scaling of the same neuropils (*SI Appendix*, Fig. S9 and Dataset S4*A* and S4*B*). After controlling for covariance between these three physically and functionally connected neuropils, significant effects of EC1 and EC2 were retained for the lobula plate (MCMCglmm: EC1, *P*-mean = 0.010, 95% CI = 0.004 to 0.016, *P*_MCMC_ = 0.001; EC2, *P*-mean = 0.011, 95% CI = 0.003 to 0.019, *P*_MCMC_ = 0.007; Dataset S4*A*), suggesting that selection shaping behavioral processes known to be primarily mediated by this structure (e.g., flight maneuverability) might particularly explain volumetric shifts in optic lobe investment (44).

Similar associations were not found for sensory neuropils within the central brain, including the antennal lobe, the primary olfactory processing center, implying that greater investment in visual processing does not correlate with reductions in olfactory investment, or vice versa (45) (Fig. 4*D* and *SI Appendix*, Fig. S8 and Dataset S4*A* and S4*B*). Previous data showing extensive visual system variation from insectary-reared individuals of four ithomiine species also suggests that these shifts are likely heritable, rather than the result of environmentally induced plasticity (34, 46). Our results therefore demonstrate that both peripheral and central components of the visual pathway vary in size nonallometrically, which is highly suggestive of selection to increase their capacity to exploit visual information based on the abundance and reliability of visual cues in their preferred microhabitat. This likely reflects a balance between the energetic costs of visual investment and quality of visual information gained through that investment, which together determine how visual systems are optimized among diurnal terrestrial species (3, 47). This contrasts with some previously reported patterns of evolution in Lepidoptera where visual systems evolve to maximize the capture of unfavorable light abundance, as is seen during transitions to nocturnality in many species (e.g., ref. 48), suggesting that selection to maximize light capture in light limited microhabitats is not the primary determinant of variation in the visual system of ithomiines. Although the behavioral consequences of these visual system differences remain to be clarified, evidence from other Neotropical butterflies have confirmed that shifts in sensory investment can significantly influence how species respond to, and prioritize, sensory stimuli (49, 50). Convergent sensory adaptations to light environments could have also evolved to exploit specific visual cues from host plants, which are also known to be more similar among comimetic ithomiines in some communities (24, 51). Indeed, it seems highly plausible that variation in light conditions between microhabitats would directly impact the distribution of plant species, which could lead to increased overlap in host plant exploitation. However, the visual cues used by ithomiines when foraging, and how similar these are across host plant species within and between microhabitats, are not known. Available data on ithomiines also suggest that they have increased reliance on chemical cues when finding plant resources (52, 53), and we find no evidence for convergent shifts in antennal lobe investment in comimetic species (54, 55). Moreover, studies in other Neotropical butterflies have found no associations between these features and large-scale structural changes in visual systems (46). As such, a direct link between variation in host plant use and sensory system convergence seems unlikely.

### Variation in Light Environments Drives Sensory Convergence Among Comimics.

In Ithomiini, and other radiations of mimetic butterflies, shifts in mimicry patterns are known to instigate speciation processes, with ecological segregation accelerating reproductive isolation (30–32). Evidence from closely related species or populations distributed along environmental gradients also strongly suggests that divergence in visual system architecture may reinforce ecological divergence (15–18). As such, secondary local adaptation across distinct environmental conditions may be a contributing factor in creating and maintaining community diversity among closely related species. Given our evidence that multiple aspects of variation in the visual system are associated with light environments, we next tested whether convergence in light microhabitat preference within ithomiine mimicry rings could also predict adaptive, interspecific convergence in sensory traits. We approached this through multiple statistical methods. First, when data from individual butterflies were analyzed using phylogenetic generalized linear mixed models, which control for species identity and evolutionary relatedness, the addition of the three main mimetic clusters (three groups: tiger-stripe, clearwing, confusa) improved model fit in all measured visual traits, except for the relative abundance of red-reflecting facets (Figs. 3 and 4 and Dataset S5). The fit of these models was also better than alternative models that included the eight mimicry ring groupings as a fixed effect instead, while producing consistent results (*SI Appendix*, Fig. S10 and Dataset S5). Standardized major axis regressions also confirmed that these differences were the result of nonallometric adaptive “grade shifts” (shifts along the y-axis) between mimetic clusters, except for the lobula which showed a significant shift in the allometric slope (Figs. 3 and 4 and Dataset S5). These results were further supported by a significant effect of mimetic cluster on all visual traits when species means were analyzed with phylogenetic generalized least squares regression (PGLS), with the exception of eye surface area and facet number (Dataset S5).

**Fig. 5.**
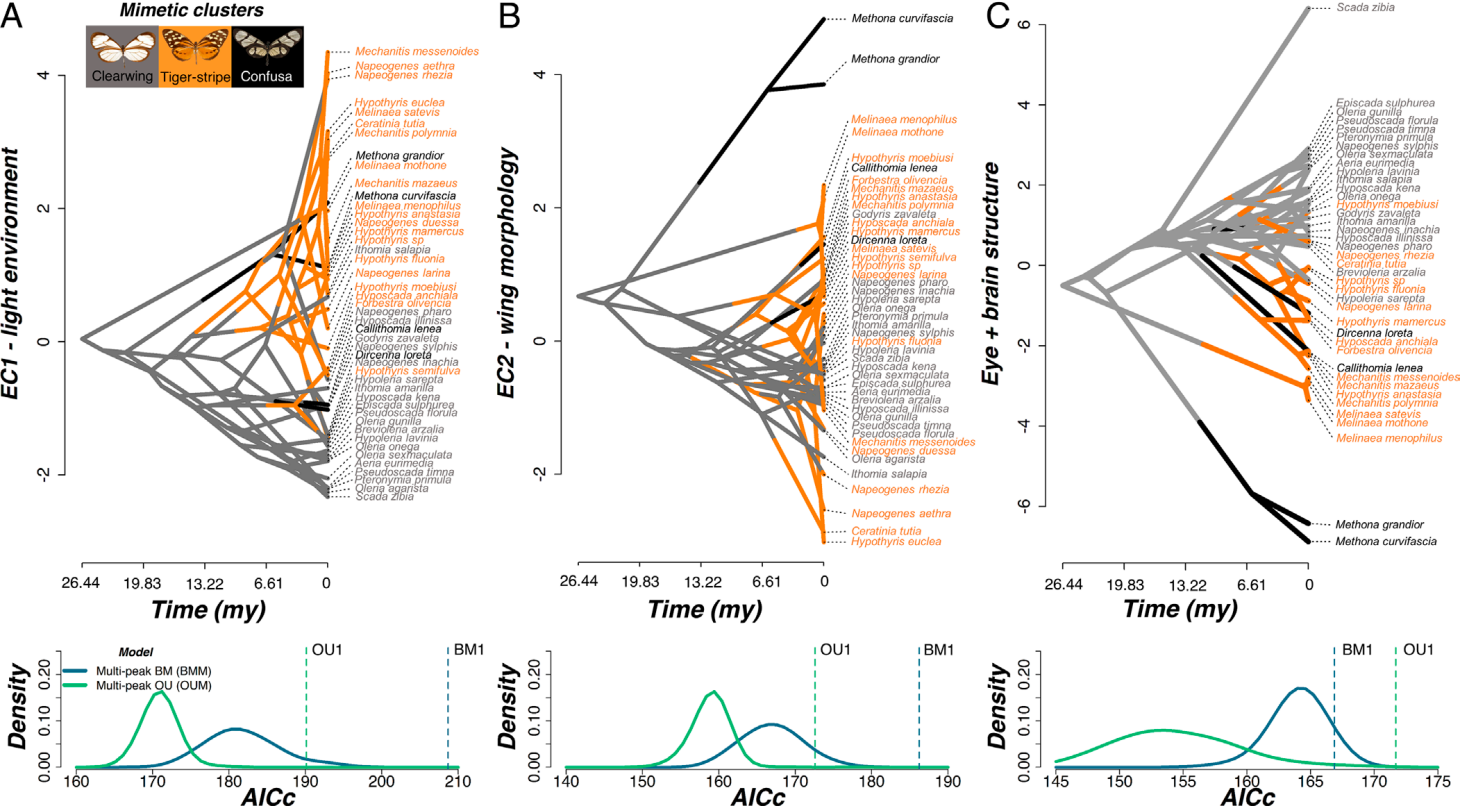
Adaptive convergence in light environment, wing morphology, and visual system structure among comimics. (*A*–*C*) Phenograms (*Top*) based on EC1, EC2 (*A* and *B*) (*N* = 45 species), and PC1 of a PCA which summarized variation in eye and brain structure based on traits which showed significant light environmental effects (*C*) (*N* = 40 species). Instances where branches cross and concentrate in a given area indicate convergent lineages. Below each phenogram are Kernel density plots of small sample corrected Akaike Information Criterion (AICc) scores obtained from multipeak BM and OU evolutionary models, constructed from 500 simulated character maps where species belonging to the same mimetic cluster were assigned to the same selective regime. Blue and green vertical dashed lines indicate the AICc score for single-peak BM (BM1) and OU (OU1) models respectively. Example models of each mimetic cluster are shown in the *Top Left* of *A*.

These findings demonstrate that i) mutualistic interactions among Müllerian mimics lead to the partitioning of forest light microhabitats, most likely an effect driven by contrasting signal efficiencies of warning cues to predators; and ii) this microhabitat partitioning predicts the sensory selection regime a species experiences, and favors the evolution of sensory convergence within mimicry rings, and sensory divergence between mimicry rings, due to contrasting selection regimes in different light microhabitats. To further test whether this convergence arose via adaptive evolutionary processes, we summarized variation in traits that showed significant nonallometric light environmental effects (relative abundance of red-reflecting facets, eye surface area, facet number, facet diameter, and the volume of the medulla, lobula plate, and lobula, and rest of central brain volume as an allometric control) along a single principal component axis (which explained 78.95% of variation in the data). Although this axis might capture allometric and nonallometric components of trait variation, the loading of our allometric control (rest of central brain volume) along this axis is relatively low compared to other traits (Dataset S6) suggesting nonallometric differences are a more distinguishing feature of visual structural variation. With this summary component, which reflects variation across the visual pathway and correlates independently with both EC1 and EC2 (PGLS, lambda = 1.000; EC1, t = −5.864, *P* < 0.001; EC2, t = −7.768, *P* < 0.001), we constructed single and multipeak Brownian motion (BM), Ornstein–Uhlenbeck (OU), and early burst (EB) models of evolution (*SI Appendix*, *SI Methods*). EC1 (light environment) and EC2 (flight-related wing morphology) were also modeled in the same way. Interspecific variation in light environment preference, flight-related wing morphology, and variation in the visual pathway, consistently fitted multipeak OU models above all other models, indicating that species in the same mimetic cluster are attracted toward convergent adaptive optima (Fig. 5 and Dataset S7). Visual system convergence was further supported by equivalent models constructed for eye and brain structures separately (using separate eye and brain PC axes) and by additional tests for evolutionary determinism (*SI Appendix*, Fig. S11 and Dataset S7). Together, these analyses provide strong evidence that directional evolutionary change, associated with convergence in both light microhabitat and flight-related wing morphology, has contributed to similarities in a suite of visual system traits, reflecting local adaptation. The mutually beneficial interactions between comimetic species, a classic example of interspecific mutualism (23, 28, 29), therefore indirectly shapes the adaptive evolution of visual traits toward predictable adaptive peaks. This creates convergence among species which belong to the same mimicry ring, and divergence between species belonging to distinct mimicry rings. Where closely related species diverge into distinct mimicry rings, divergent selection on visual systems may therefore contribute to the suite of traits that together shape and maintain ecological isolation.

## Summary

Our study provides empirical evidence that relatively small shifts in terrestrial light environment properties can shape adaptive and predictable visual system evolution between sympatric species, over very small spatial scales—a phenomenon only previously hypothesized (34, 35). Our data also offers insights into how variation in ecologically relevant light environments shape the assemblage of diverse communities. Specifically, we have shown that light abundance and spectral composition within rainforests can contribute to multiple dimensions of niche separation that overcome phylogenetic niche conservatism. We have further demonstrated how these fluctuations in ambient light conditions can be a potent selective force, predictably driving visual systems toward distinct adaptive peaks. Notably, our data also reveal how mutualistic mimetic interactions can have broad-reaching and perhaps surprising effects on organismal evolution. In this case, the mutualistic interactions among comimics not only drive divergence in wing pattern (24, 25), flight behavior (26), and microhabitat preference (20, 21), but by doing so it indirectly alters selection regimes acting on sensory systems, impacting multiple levels of visual pathways. We conclude that both ambient light and biotic interactions (i.e., Müllerian mimicry) play a crucial role in shaping both tropical forest communities and patterns of visual system evolution. Together, this introduces mimetic butterflies as an informative model system for disentangling the many manifestations by which species have evolved to perceive and process their sensory world.

## Methods

### Field Site, Animal Collection, and Identification.

All fieldwork was conducted along designated trails which surround the Estación Científica Yasuní (0°40′27″ S, 76°23′50″ W), at Parque Nacional Yasuní, Orellana Province, Ecuador, a ~4.5 km^2^ area of primary lowland Amazon rainforest where local ithomiine diversity is high (~60 recorded species) (20). All ecological measurements and eye samples were taken in August–October 2022 between 8:00AM (08:00 h) and 2:00PM (14:30 h), when butterflies are most active, under permit collection no. MAAE-ARSFC-2021-1763 and export permit no. 023-2022-EXPIC-FAU-DBI/MAAE. Brain samples were collected in November/December 2011 and September/October 2012 under permit collection no. 0033-FAU-MAE-DPO-PNY and export permit nos. 001-FAU-MAE-DPO-PNY and 006-EXP-CIEN-FAU-DPO-PNY. Permits were obtained from Parque Nacional Yasuní, Ministerio del Ambiente, La Dirección Provincial de Orellana, with support from the Pontificia Universidad Católica del Ecuador (PUCE) and staff at the Estación Científica Yasuní.

Studying a single diverse community allows for robust comparative analysis between species and mimicry rings by eliminating variation due to geographical variables such as altitude and climate. Collecting data across three ~2-mo field seasons also meant that the relative species abundances in our datasets were representative of their natural numbers in the community. Across the three field seasons, 54 ithomiine species were sampled pertaining to the eight local mimicry rings (agnosia, aureliana, confusa, eurimedia, hermias, lerida, mamercus, and mothone; Fig. 2*A*) (20, 21), and nine of the ten major ithomiine subtribes (Dircennina, Godyridina, Ithomiina, Mechanitina, Melinaeina, Methonina, Napeogenina, Oleriina, Tithoreina) (19, 24). Previous studies have shown these eight mimicry rings are spatially segregated across microhabitats, defined by vegetation type and height, and through predictable shifts in flight height (20, 21). Genera were identified using wing venation patterns (56) and then identified to species level using ID sheets for the races found locally at Yasuní, provided by Dr Keith Willmott (University of Florida), and sexed. The wings of all sampled individuals were kept in transparent envelopes as voucher specimens. Body length (cm) was also measured as a condition-independent measure of body size. In 2022, a maximum of twelve individuals per species were sampled for eye physiological and anatomical analysis. Nonretained individuals were IDed, sexed, and marked on one wing with black permanent marker prior to release to avoid future resampling.

### Spectral and Ecological Data.

#### Transect measurements.

To investigate whether changes in forest structure created distinct light environments, spectral measurements were obtained at designated points along the “Mirador” trail, a relatively straight 2.1 km topographically variable transect consisting of nine consecutive ridges (~250 m above sea level) and valleys (~240 m above sea level) (Fig. 1). Ten spectral measurements were taken at each ridge/valley replicate at four height categories (1, 2, 3, and 4 m) which were chosen based on flight height data for ithomiines previously reported by Willmott et al. (33) (Fig. 1). Time of day was also recorded and later binned as “early morning” (08:00 to 09:59 h), “late morning” (10:00 to 11:59 h), and “early afternoon” (12:00 to 13:59 h). Individual ithomiine butterflies caught along this transect were assigned to the nearest ridge or valley and one of the four height categories (1, 2, 3, and 4 m), as in ref. 33. No butterflies were sampled above 4 m. Although this may have created a sampling bias, other studies have shown ground surveys to provide an accurate proxy of ithomiine vertical stratification and, in practice, very few butterflies were observed flying above 4 m, which was still comfortably within the maximum reach of the hand nets (~5.5 m) (20, 24, 33).

Spectral irradiance measurements were taken using a lightweight, portable, open source, spectroradiometer system (OSpRad), developed by Troscianko (57), consisting of a Hamamatsu C12880MA microspectrometer chip (spectral range of 310 to 880 nm, 288-site CMOS sensor, minimal spectral resolution of 9.7 nm), combined with an automated shutter (controlled by a digital servo, Savox SH-0256) and cosine-corrector, and an Arduino Nano microcontroller, contained within custom made, 3D printed ABS plastic housing. OSpRad comes with a custom-built app written in Python and was run via the Pydroid 3 app installed onto a CUBOT Quest Lite smartphone (Android 9.0). Spectral calibration was performed using a full-spectrum xenon light source (Neewer NW-14EXM) with irradiance measures taken using a Jeti Specbos (1211UV) spectroradiometer with NIST-traceable calibration. OSpRad provided a measure of ambient light intensity from all light sources illuminating the spectroradiometer sensor for each of the 288 sensor photosites (irradiance measured in Wsr^−2^m^−2^nm^−2^; mean integration time: 14.8 ms; no. of scans per measurement: 50; Fig. 1*B*). For measurements taken at 3 and 4 m, the OSpRad system was attached to the end of hand net poles and raised to the appropriate height.

Hemispherical photography was also used to estimate canopy openness at each ridge/valley replicate by taking an upward facing photograph with the 13 MP camera of a CUBOT Quest Lite smartphone (Android 9.0) attached to a 180° clip-on fisheye lens (Fig. 1*A*). JPEG images were run through an adapted version of the *hemispheR* R package (58, 59). The blue channel was extracted from the original RGB images as this provided the best contrast between sky and canopy. Thresholding was then performed using the binarize_fisheye function (using the “Otsu” method), producing binary images analyzed by the gapfrac_fisheye function which calculates the gap fraction for each image within the zenithal angle range of 0 to 70° using seven zenith angle rings and eight azimuth segments. Canopy openness (%) was then estimated using canopy_fisheye based on the angular distribution of each gap fraction.

#### Individual measurements.

Alongside the transect measurements, individual light, and ecological measurements (including hemispherical photography) were taken for 785 butterflies across 45 ithomiine species found along all trails surrounding the Estación Científica Yasuní. Species-specific sample sizes varied from 65 for *Hypothyris anastasia* to 1 for *Hypothyris semifulva*, *Ithomia salapia,* and *Napeogenes aethra* with 24 species consisting of more than twelve individuals. Flight height was estimated for each butterfly using tape measures and the known length of hand net poles. Most butterflies were caught within arm’s reach (~3 m) but for those that were not, the OSpRad system was attached to the end of hand net poles and raised to the approximate location of initial observation, using distinct features of forest layers and landmarks as reference points.

#### Visual modeling.

Irradiance measurements taken by OSpRad were modeled based on the trichromatic visual system of *Danaus plexippus* (Nymphalidae: Danainae), the most closely related species to the Ithomiini for which the maximal sensitivity (λ_max_) of each visual pigment is known (λ_max,,_ UV = 340 nm, B = 435 nm, LW = 545 nm) (43). Opsin sequence analysis by Wainwright et al. (34) suggests that ithomiines possess the same number of functional photoreceptors as *D. plexippus*, making *Danaus* a suitable model visual system for calibrating the spectral measurements (see *SI Appendix*, *SI Methods* for details).

A measure of overall photon catch was calculated as the estimated quantum catch of the long-wavelength-sensitive photoreceptor, the primary achromatic channel in invertebrates (36). As an alternative, the mean quantum catch of all three spectral channels (UV, B, and LW) was also calculated. Both measures of photon catch were log_10_ transformed prior to analysis and analyzed in the same way. To quantify spectral composition, Michelson contrasts were calculated between each spectral channel (*A*) and the summed average of the remaining two spectral channels (*B* and *C*) ((*Q_a_* – *Q*_(*b*+*c*)/2_)/(*Q_a_* + *Q*_(*b*+*c*)/2_)), providing an empirical measure of relative ultraviolet, blue, and long-wavelength catch (see *SI Appendix*, *SI Methods* for additional visual modeling analyses).

#### Statistical methods.

To test whether the visual environment varied along the transect according to topography, canopy openness, and height from the ground, linear mixed models were constructed using the function lmer in the *lme4* package in R (59, 60). Both measures of overall photon catch, PC1-3, and relative UV/B/LW catch were each regressed against topography, height (as an ordinal factor), canopy openness, and their interaction. Replicate number, day, and time of day were included as random effects. The significance of each fixed effect was determined by comparing models with and without the variable in question using the *anova()* function.

Based on the results of the above models and previous findings of ecological segregation between ithomiine mimicry rings (20), we then sought to test whether the abundance of encountered mimicry rings differed between forest microhabitats which displayed significant variation in light environment. This was achieved by constructing Bayesian phylogenetic generalized linear mixed models with the R package *MCMCglmm* (function MCMCglmm, family = “categorical” for binary response variables, family = “Gaussian” for continuous, parametric response variables) (61) using the inverse correlation matrix of a calibrated and pruned ithomiine phylogeny from Chazot et al. (19) (packages *phytools* and *ape*) (62, 63). Default priors were used as fixed effects and uninformative, parameter expanded priors as random effects (G: V = 1,n nu = 1, alpha.mu = 0, alpha.V = 1,000; R: V = 1, nu = 0.002), as in Wainwright and Montgomery (35). Species and sex were always included as random effects and models were run for 5,100,000 iterations with a burn-in of 100,000. We report the difference in deviance information criterion (ΔDIC) with and without mimicry ring, where lower DICs indicate a better fit and models with ΔDIC < 2 are considered equivalent.

We also performed tests for mimetic segregation between light environments using spectral and ecological measurements obtained from individual butterflies. By applying the same parameters as described above, MCMCglmms were built for each spectral variable with mimicry ring, flight height, canopy openness, and the interaction between these variables included as fixed effects. To assess the significance of continuous variables (flight height and canopy openness), we report the posterior mean (*P*-mean), the 95% CIs, and the *P*_MCMC_.

By calculating species means for each variable of interest, we also recreated all of the above MCMCglmm models using a phylogenetic generalized least-squares regression (PGLS) by implementing the *pgls* function in the *caper* R package (64) with Pagel’s λ set to 1 to allow conservative comparisons between models using *anova*(). In this dataset, the polymorphic species *M. messenoides* was assigned to the hermias mimicry ring as this was the observed mimicry pattern for the majority of wild-caught individuals (70.59% hermias, 29.41% mothone).

### Summarizing Ecological Variation.

To disentangle the relative roles of light environment and flight-related morphologies in driving visual system evolution (see main text), forewings from individuals sampled in 2011/2012 were photographed dorsally using a DSLR camera and 100 mm macrolens under standardized lighting conditions. Damaged forewings were excluded from the analyses. By running adapted custom scripts from Montejo-Kovacevich et al. (65) in FIJI (66), wing surface area (mm^2^) and aspect ratio were calculated from these images. The latter was defined as the ratio of the major and minor wing axes’ lengths and was used as a proxy of wing shape, which is known to predict flight speed in many butterfly species (41). Wing loading (g mm^−2^) is known to positively predict the lift required to fly at the desired speed and was thus also calculated, by dividing body mass (in this case, the total mass of the head, thorax, and abdomen) by forewing area.

The species means of all wing morphological variables (forewing surface area, aspect ratio, forewing loading) and ecological variables (flight height, canopy openness) were included in a PCA along with the mean quantum catch of the UV, B, and LW spectral channels. The loadings from this PCA revealed all variation to be summarized along two axes; the light environmental axis (EC1, 45.99% explained variation) and the wing morphological axis (EC2, 28.71% explained variation) (eigenvalue cutoff = 1) which were used in subsequent analyses (see main text; Dataset S3). Mean canopy openness and flight height loaded positively on EC1 and EC2 respectively. Mimetic segregation along both these axes was investigated using PGLS (Spectral and Ecological Data: Statistical Methods).

### Physiological and Anatomical Trait Data.

#### Eye physiological measurements.

Eye physiology was studied at the Estación Científica Yasuní at room temperature using live, wild-caught individuals (*N* = 363, 56.25% female, average of ~8 individuals per species) with a custom-built ophthalmoscope, previously described by Wainwright et al. (34), connected to a laptop with the uEye Cockpit program (IDS Software Suite 4.95) installed (see *SI Appendix*, *SI Methods* for details). We imaged the luminous pseudopupil, a region where the optical axes of several ommatidia are aligned and emit colorful eyeshine after dark adaptation (67, 68). Pupillary response time and the presence of red-reflecting facets were measured (*SI Appendix*, *SI Methods*). The number of reflecting facets were counted and categorized as being yellow or red, from which the ratio of yellow:red reflecting facets was calculated for each individual. The presence of red screening pigments was confirmed in at least one individual of each species.

#### Eye anatomical measurements.

Following ophthalmoscopy, heads were removed and preserved in chilled RNAlater (Invitrogen^TM^ AM7021 ThermoFisher Scientific), alongside the remaining body tissue for future molecular work. Samples were kept at 4 °C whenever possible before being returned to the United Kingdom where they were stored at −20 °C. Whole heads were imaged (proboscis and labial palps removed) from the frontal view with LAS X software using a Leica EZ4 W stereo microscope with an integrated 5 MP camera at 20× magnification (Fig. 3*C*). One compound eye was removed using a fine blade and forceps and placed into 20% sodium hydroxide solution for 3 to 5 h to loosen the ommatidia and underlying pigment from behind the eye cuticle. The eye cuticle was then isolated before making four small cuts, two on the dorso–ventral axis and two on the posterior–anterior axis, enough so that it could lay flat. The flattened cuticle was cleaned of debris under 95% ethanol solution and mounted on a clean microscope slide with a small drop of Euparal (Elkay Laboratory Products Ltd) under a cover slip. Mounted slides were then left for a minimum of 24 h before being imaged with GXCapture software on a Leica M205 C stereo microscope with an integrated 8 MP camera. Images were taken at 1.6 ×, 2.0 ×, 2.5 ×, 3.2 ×, and 4.0 × magnification depending on the size of the sample (Fig. 3*C*).

TIFF images of whole heads were imported into FIJI/ImageJ where interocular distance (mm), defined as the minimum horizontal gap between the two eyes, was measured using the line tool. After correcting the scale in FIJI/ImageJ, facet number, eye surface area, and mean facet diameter were measured from cuticle images using the ommatidia detecting algorithm (ODA) (69) (Fig. 3*C*). This module, written in Python, identifies individual facets from 2D images by extracting periodic signals from each image using a 2D fast Fourier transform. All anatomical variables were log_10_ transformed before any statistical analysis.

#### Sensory neuroanatomical measurements.

Relative investment in sensory processing structures was investigated using a separate sample of wild-caught ithomiines (collected in 2011 and 2012) consisting of 392 individuals across 49 species. Species sample sizes varied from 28 for *H. anastasia* to 1 for *Callithomia alexirrhoe*. *Ceratinia tutia*, *Dircenna loreta*, *Heterosais nephele*, *Ithomia agnosia*, *Pteronymia primula, Pteronymia vestilla*, and *Tithorea harmonia*, with 17 species consisting of more than eight individuals (*SI Appendix*, Fig. S3*B*). The neuropil volumes within this dataset include those from individuals previously acquired and analyzed by Montgomery and Ott (54) and Wainwright and Montgomery (35) which were collected during the same two field seasons. The brain tissue of the remaining samples were preserved and subsequently stained following an established protocol, using anti-SYNORF as a primary antibody, Cy2 as a secondary, and imaged on a confocal laser-scanning microscope (see *SI Appendix*, *SI Methods* for details). Neuropils were then segmented manually in Amira 3D analysis software 2021.2 (ThermoFisher Scientific, FEI Visualization Sciences Group) (*SI Appendix*, *SI Methods*). This procedure was used to reconstruct the volume (μm^3^) for five of the six primary optic lobe neuropils (medulla, lobula plate, lobula, accessory medulla, and ventral lobula with total optic lobe size being calculated from the sum of these raw volumes). The lamina was not included as it is extremely thin in many smaller species and was easily damaged during the dissections, so could not be obtained reliably for the full range of species. The ventral lobula, a small neuropil found in the optic lobe, was absent in many individuals, particularly females (34). Volumes were also reconstructed for the anterior optic tubercle, as the most prominent central brain visual structure, and antennal lobe, as the primary olfactory processing center, to test for comparable effects on olfactory investment. Raw volumes for the anterior optic tubercle and antennal lobe were subtracted from a measure of central brain volume to calculate the rest of central brain which acted as an allometric control in subsequent statistical models (Fig. 4*A*). Each paired neuropil was multiplied by two and all volumes were log_10_ transformed before any analysis.

#### Statistical methods: Regression analyses.

To test whether variation in all our measured visual traits correlate with light microhabitat preference, we regressed each visual trait against EC1 (again, using MCMCglmm; *Spectral and Ecological Data: Statistical Methods*). EC2, its interaction with EC1, and, where necessary, a relevant allometric control were included in the same model as additional fixed effects (see *SI Appendix*, *SI Methods* for additional analyses). We also tested for covariance among physically and functionally connected neuropils in the brain by constructing separate MCMCglmms where neuropils, whose volume showed a significant nonallometric association with either EC axis, were regressed against all other significant neuropils.

For physiological and anatomical traits which demonstrated evidence of light environment convergence, in both the MCMCglmm and PGLS analysis, we then tested whether mutualistic interactions between species, as indicated by mimicry pattern, could result in downstream shifts in these traits across an entire community of ithomiine species (again, using MCMCglmm and PGLS), expanding on more focused comparisons previously conducted within this tribe (34, 35). For these analyses, mimicry rings were regrouped based on how species were segregated between light environments. Additional models were also constructed with the original mimicry ring classifications as independent variables. As a post hoc to the above analysis, we conducted additional tests on anatomical traits which showed convergence with mimicry (in either the MCMCglmm or PGLS analysis), using the sma function in the *smatr* package (70). This function detects pairwise shifts in the scaling relationship between the trait of interest and the appropriate allometric control, by testing for slope (β shift), elevation (α or grade shift), and major axis shifts.

#### Statistical methods: Evolutionary modeling and analysis of convergence.

We used models of morphological evolution to investigate changes in evolutionary dynamics of visual traits that showed evidence of convergence in light environment. We focused on BM and OU models, implemented in the R package *mvMORPH* (71). BM models represent random-walk evolutionary processes where the evolutionary rate (parameter σ^2^) is constant. OU processes include both a stochastic BM component and a deterministic tendency toward an optimal value (*θ*), governed by the strength of selection (α) toward adaptive optima, the optimum being the average phenotype toward which lineages subjected to the same “selective regime” have evolved. In the present case, these selective regimes were predefined as the mimetic cluster to which a species belonged. Our hypothesis was that comimics, or those sharing similar light environments, will have evolved toward convergent adaptive optima in sensory anatomical trait space (see *SI Appendix*, *SI Methods* for details).

## Supplementary Material

Appendix 01 (PDF)

Dataset S01 (XLSX)

Dataset S02 (XLSX)

Dataset S03 (XLSX)

Dataset S04 (XLSX)

Dataset S05 (XLSX)

Dataset S06 (XLSX)

Dataset S07 (XLSX)

## Data Availability

All raw data, R analysis code, uncalibrated spectral data, visual modeling templates, hemispherical photography images, forewing images, eyeshine video recordings, eye cuticle images and analyses’ output, confocal microscopy image stacks, and 3D brain reconstruction files are available from Zenodo (72): https://zenodo.org/records/12759660.
