# Supplementary material for: Mutualisms within light microhabitats are associated with sensory convergence in a mimetic butterfly community
Source: Proc Natl Acad Sci U S A. 2025 Jul 15;122(29):e2422397122. doi: 10.1073/pnas.2422397122 (PMC12305024; doi:10.1073/pnas.2422397122)
Supplement: Supplementary file 1 — Appendix 01 (PDF) [file pnas.2422397122.sapp.pdf]

Supporting information for:

Title: Mutualisms within light microhabitats are associated with sensory convergence in a mimetic butterfly community

Authors: J. Benito Wainwright[1]*†, Theodora Loupasaki[1], Francisco Ramírez[2], Iestyn L. Penry Williams[1], Sam J. England[3], Annalie Barker[4], Joana I. Meier[4,5], Martin J. How[1], Nicholas W. Roberts[1], Jolyon Troscianko[6], Stephen H. Montgomery[1]*

Affiliations:

[1]School of Biological Sciences, University of Bristol; Bristol, UK.

[2]Museo de Zoología QCAZ, Laboratorio de Entomología, Escuela de Ciencias Biológicas, Pontificia Universidad Católica del Ecuador, Quito, Ecuador.

[3]Department of Evolutionary Morphology, Museum für Naturkunde-Leibniz Institute for Evolution and Biodiversity Science; Berlin, Germany.

[4]Department of Zoology, University of Cambridge; Cambridge, UK

[5] Tree of Life Programme, Wellcome Sanger Institute; Hinxton, UK.

[6]Centre for Ecology & Evolution, University of Exeter, Penryn Campus; Penryn, UK.

*Corresponding authors. Emails: jbw21@st-andrews.ac.uk, s.montgomery@bristol.ac.uk

†Present address: Centre for Biological Diversity, School of Biology, University of St Andrews, Fife, UK.

SI Methods

Visual modelling details

The λ_{max} values of the ultraviolet (UV), blue (B), and long-wavelgnth (LW) visual pigments from *Danaus plexippus* were used to estimate absorbance spectra with nonlinear least-square fitting according to the template by Govardovskii *et al.*[1] (Fig. S1). Although this template was originally designed to model the spectral sensitivity of vertebrate visual pigments, previous studies have shown that it is practically identical to invertebrate models at accurately describing the normalized sensitivity of rhodopsins, particularly within the visible range[2]. The absorbance values from these models, and the integration time of each spectral reading, were used to calibrate the raw irradiance measurements. This calculated photoreceptor photon capture (quantum catch, *Q*) for the ultraviolet (UV), blue (B) and long-wavelength (LW) sensitive spectral channels, providing a measure of how many photons are absorbed by each photoreceptor under the given lighting conditions (measured in 10^{10} quanta s^{-1} m^{-2}).

It is worth noting that *Danaus,* and likely Ithomiini, also contain a fourth long-wavelength sensitive spectral channel created by the presence of red screening pigment, as indicated by red-reflecting ommatidia in their eyeshine[3,4]. However, the relative contribution of these screening pigments in shifting the spectral sensitivity of photoreceptors is difficult disentangle, and the inter-ommatidial connections underlying color processing is poorly understood, making visual modelling using current methods difficult[4,5]. Our estimated quantum catches are therefore a conservative estimate of the visual information available to an ithomiine butterfly within its respective microhabitat.

Additional visual modelling analyses

Two principal component analyses (PCA) were also conducted for the transect and individual light measurements respectively. In both PCAs, PC1 explained, 98-99% of variation in quantum catch, receiving equal loadings from all three spectral channels, indicating that this is achromatic and equivalent to our measure of overall photon catch (Dataset S1,S2). The remaining variance summarized by PC2 and PC3 describe chromatic variation with the UV channel loading negatively against B and LW for PC2, and the B channel loading negatively against UV and LW for PC3. This suggests opponency between these channels, which complements the mechanisms by which colors are processed in other insects (e.g. [6,7]). To satisfy the model assumptions of our downstream analyses, the PC scores were normalized by $\log_{10}(n + 2)$ and $\log_{10}(n + 1)$ transformation for the transect and individual measurements respectively, to avoid log-transformation of negative values. Analyses of these PC axes generally mirrored results from overall photon catch and relative wavelength catch analysis, as well as in models where PC scores were not transformed (for full details see Dataset S1,S2).

Eyeshine recordings

Butterflies were mounted in slotted plastic tubes, immobilized using plasticine, and oriented with a micromanipulator to face the objective lens of a custom-made ophthalmoscope (see Wainwright *et al.* [3]), so the frontal region of the compound eye was in view. The ophthalmoscope was then adjusted to focus on the luminous pseudopupil, a region where the optical axes of several ommatidia are aligned and emit colorful eyeshine after dark adaptation, due to the presence of a tapetal reflector at the proximal end of each rhabdom[8]. The color and heterogeneity of the eyeshine varies between butterfly species but its intensity diminishes

quickly upon exposure to light due to intracellular pigment migration towards the rhabdomeres, preventing incoming light from reaching the tapetum[4,9]. Video recordings of the eyeshine were taken after five minutes of dark adaptation under standard laboratory conditions and imported into FIJI/ImageJ[10] where pupillary response time and the ratio of yellow:red reflecting facets was extracted from each video. The speed of the pupillary response is known to vary between ithomiine species, suggesting that it might reflect how eyes physiologically respond to spatial and temporal variation in the light conditions present within their respective microhabitats[3]. The presence of red-reflecting facets in the eyeshine is indicative of screening pigment which is known to create an additional long-wavelength spectral channel in some nymphalid butterflies, the existence of which varies both between and within species (e.g. [4,11]). We focus on these data as available molecular evidence suggests general conservation of all functioning photoreceptors in ithomiines, which means shifts in eyeshine color are more likely to be a result of changes in screening pigment expression[3].

Brain staining, imaging, and processing

Individuals were dissected at the Estación Científica Yasuní under HEPES-buffered saline (HBS; 150 mM NaCl; 5 mM KCL; 5 mM CaCl2; 25 mM sucrose; 10 mM HEPES; pH 7.4) and fixed in zinc formaldehyde solution (ZnFA; 0.25% [18.4 mM] ZnCl2; 0.788% [135 mM] NaCl; 1.2% [35 mM] sucrose; 1% formaldehyde) for 16-20 hours whilst under agitation. Samples were subsequently washed in HBS three times and placed in 80% methanol/20% DMSO for a minimum of two hours under agitation before being stored in 100% methanol at room temperature and later at -20°C upon arrival to the United Kingdom.

Brain tissue was immunostained against synapsin, a conserved protein expressed at pre-synaptic regions across insects, following the same protocols. Brains were rehydrated in a methanol-Tris buffer series of decreasing concentration (90%, 70%, 50%, 30%, and 0%, pH 7.4), for 10 minutes each, and subsequently incubated in 5% normal goat serum (NGS; New England BioLabs, Hitchin, Hertforshire, UK) diluted in 0.1 M phosphate-buffered saline (PBS: pH 7.4) and 1% DMSO (PBSd) for two hours at room temperature. Samples were stained using antiSYNORF as a primary antibody (Antibody 3C11; Developmental Studies Hybridoma Bank, University of Iowa, Iowa City, IA; RRID: AB_2315424) in NGS-PBSd, at a dilution of 1:30, and left for 3.5 days under agitation at 4°C. To remove non-bound antibody, three two-hour washes in PBSd were conducted before applying the secondary Cy2-conjugated anti-mouse antibody (Jackson ImmunoResearch; Cat No. 115-225-146, RRID: AB_2307343, West Grove, PA) at a dilution of 1:100 in NGS-PBSd. Samples were incubated for 2.5 days at 4°C under agitation before being subjected to a glycerol dilution series (diluted in 0.1 M Tris buffer, 1% DMSO) of increasing concentration (1%, 2%, and 4% for two hours each, 8%, 15%, 30%, 50%, 60%, 70%, and 80% for one hour each) and then dehydrated in 100% ethanol three times, 30 minutes each. Finally, brains were placed in methyl salicylate and left for ~30 minutes for the brain tissue to sink and clear, before being replaced with fresh methyl salicylate.

Brains were imaged at the University of Bristol's Wolfson Bioimaging Facility on a confocal laser-scanning microscope (Leica SP5-AOBS/SP5-II, Leica Microsystem, Mannheim,Germany) fitted with a 10x 0.4 NA objective lens (Leica Material No. 506285, Leica Microsystem). Each sample was scanned from the anterior and posterior side separately using a 488 nm argon laser at 20% intensity, a mechanical z-step of 2 μm, and an x-y resolution of 512x512 pixels. Anterior and posterior image stacks were later merged into a single image

stack file in Amira 3D analysis software 2021.2 (ThermoFisher Scientific, FEI Visualization Sciences Group), using a custom *advanced merge* module provided by Rémi Blanc (Application Engineer at FEI Visualization Sciences Group). Prior to image segmentation, the z voxel size of the resulting merged image stack was multiplied by 1.52, to correct for artificial shortening of the z-dimension[12]. Using Amira 2021.2, every third image of each neuropil of interest was then segmented manually, based on the intensity of the synapsin immunofluorescence, by creating label files for each individual with the *labelfield* module. All intervening unsegmented sections were then assigned to the neuropil of interest by interpolating in the z-dimension, before being edited and smoothed in all three dimensions. Volumetric information was extracted using the *measure statistics* module.

Additional visual trait analyses

When regressing visual traits against ecological PC axes, eye surface area was modelled with inter-ocular distance included as an additional fixed effect to control for allometric effects. To test whether larger eyes have evolved to optimize functional performance by increasing facet size, facet number was modelled with eye surface area as an additional fixed effect. Mean facet diameter was subsequently analysed without an allometric control to reflect differences in total light capture between species. For models with neuropil volumetric data, "rest of central brain" volume was included as an additional fixed effect to control for allometric effects. These analyses were also recreated using PGLS, as described in the main methods, with Pagel's λ being estimated based on maximum likelihood.

All visual traits were also regressed against individual spectral variables that significantly varied between ithomiine light microhabitats, using MCMCglmm and PGLS (see main

methods). Light measurements were not taken for individuals sampled for brain tissue, so species means for each spectral variable were used for the analysis of sensory neuropils. We also built separate models where visual traits were regressed against individual wing morphological variables (forewing area, aspect ratio, wing loading) to test whether patterns of visual system evolution are correlated with changes in flight-related wing morphology. Wing morphological variables were collected from individuals sampled for brain tissue only, so species means for each wing variable were used in models involving eye physiological and anatomical traits. The results from these analyses are presented in Dataset S4b.

Evolutionary modelling details

Prior to evolutionary modelling analysis, we used the species means dataset to conduct a PCA using visual traits that showed evidence of light environment convergence, plus "rest of central brain" volume as an allometric control where relevant (see main text). The significant axes from this PCA were also regressed against EC1, EC2, and their interaction, using PGLS. The reconstructed history of the selective regime on which each model was fitted, was constructed using 500 character maps in *phytools* using the 'make.simmap' function [13]. These simulated character maps were used to fit Brownian motion (BM) and Ornstein-Uhlenbeck (OU) models with single (BM1, OU1) and regime-specific optima (BMM, OUM) to univariate models (functions 'mvBM' and 'mvOU'). We also constructed an "early burst" (EB) model, where visual systems diversify rapidly early on in cladogenesis (function 'mvEB'). The fit of the resulting models was compared using the mean corrected Akaike information criterion (AICc) with lower AICc values indicating an improved model fit.

We also quantified levels of evolutionary convergence in sensory morphology between co-mimics, or those sharing similar light environments, using the C indexes (C1-4) calculated within the *convevol* package[14]. Data were simulated under Brownian motion 1,000 times to gauge significance. Lastly, a phenogram which maps visual trait evolution was created using the "phenogram" function in *phytools* where crossing branches indicate convergent evolution[13].

Supplementary Figures:

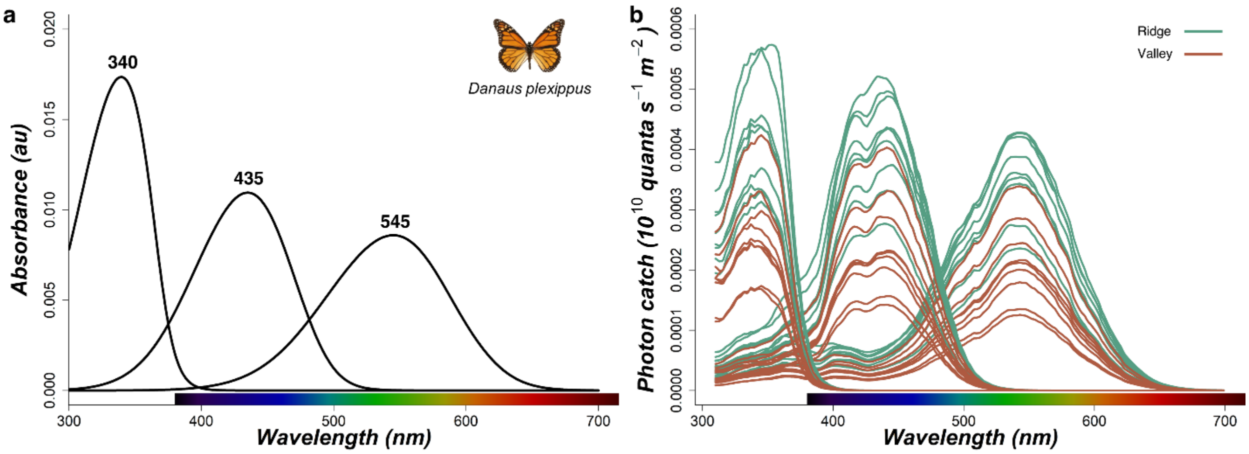

Figure S1. Modelling spectral irradiance for butterfly vision. a, Absorbance curves of the three visual pigments (from left to right: ultraviolet, blue, long-wavelength) in the adult compound eye of *Danaus plexippus* (Nymphalidae: Danainae), the most closely related species to ithomiines for which visual pigment sensitivities are known. The wavelengths of maximal absorbance (λ_{max}) of these visual pigments are labelled above the peak of each curve and were used to calibrate our raw spectral irradiance readings for butterfly vision, using the template by Govardovskii *et al.* [1]. **b**, Mean photon capture (10^{10} quanta s^{-1} m^{-2}) for ultraviolet (left peak), blue (middle peak), and long-wavelength (right peak) photoreceptors for each ridge and valley replicate along a 2.1 km topographically variable transect at the field site in Ecuador.

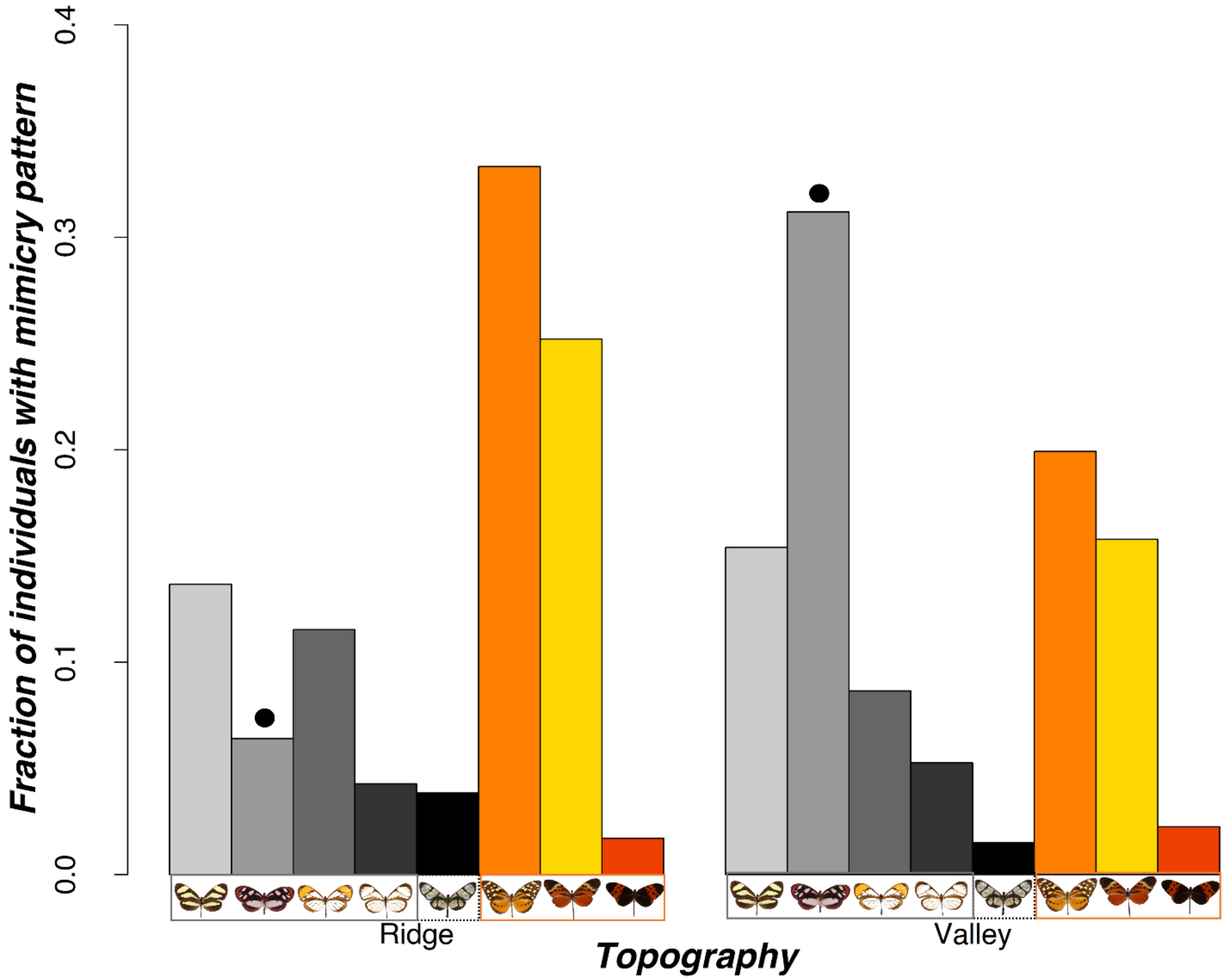

Figure S2. Relative mimicry ring abundance at ridge and valley sites along a topographically variable forest transect. Example models of each mimicry ring are shown below their corresponding bar, grouped based on their general color pattern classification (grey = ‘clearwing’, dotted black = ‘confusa’, orange = ‘tiger-stripe’). Each black dot represents a mimicry ring whose abundance significantly differed between ridges and valleys in the MCMCglmm analysis.

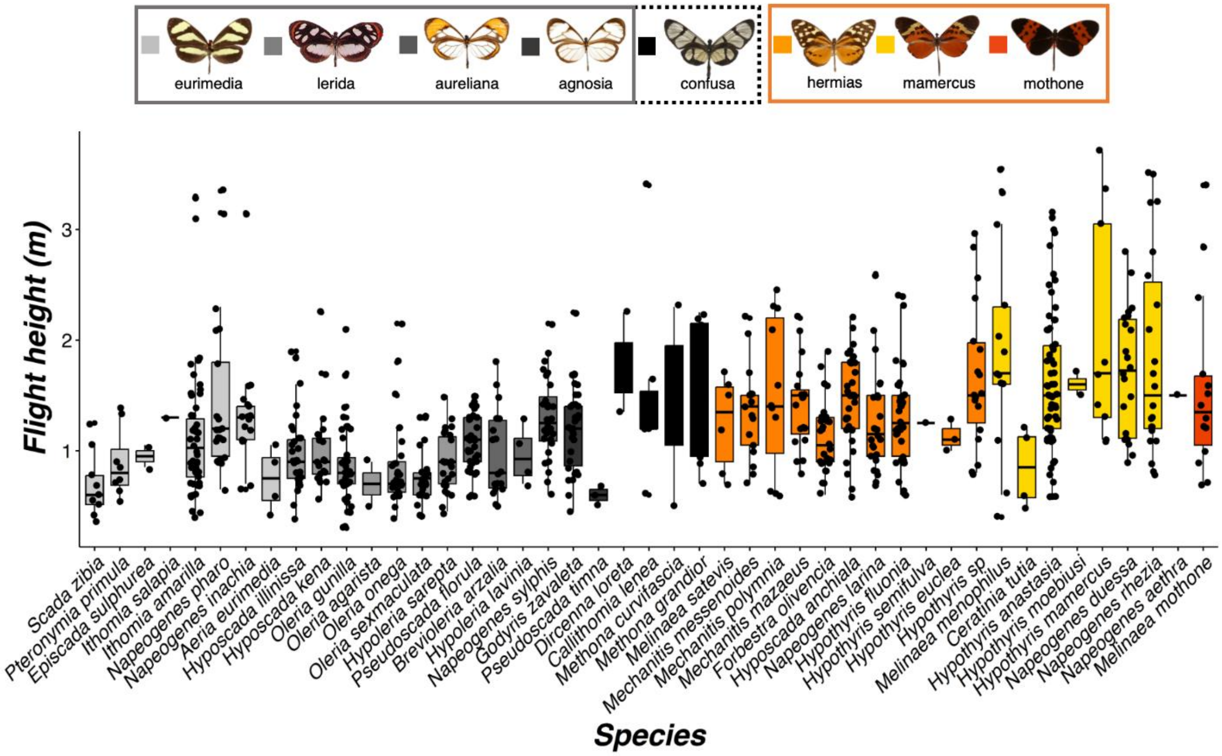

Figure S3. Flight height (m) differences between ithomiine species, coded by mimicry ring (*N* = 785, 45 species). Example models of each mimicry ring are shown on the top row, grouped based on their general color pattern classification (grey = 'clearwing', dotted black = 'confusa', orange = 'tiger-stripe'). Medians (thick horizontal bars), interquartile ranges (boxes), values within 1.5 interquartile ranges of the box edges (whiskers), and possible outliers (datapoints outside whiskers) are plotted.

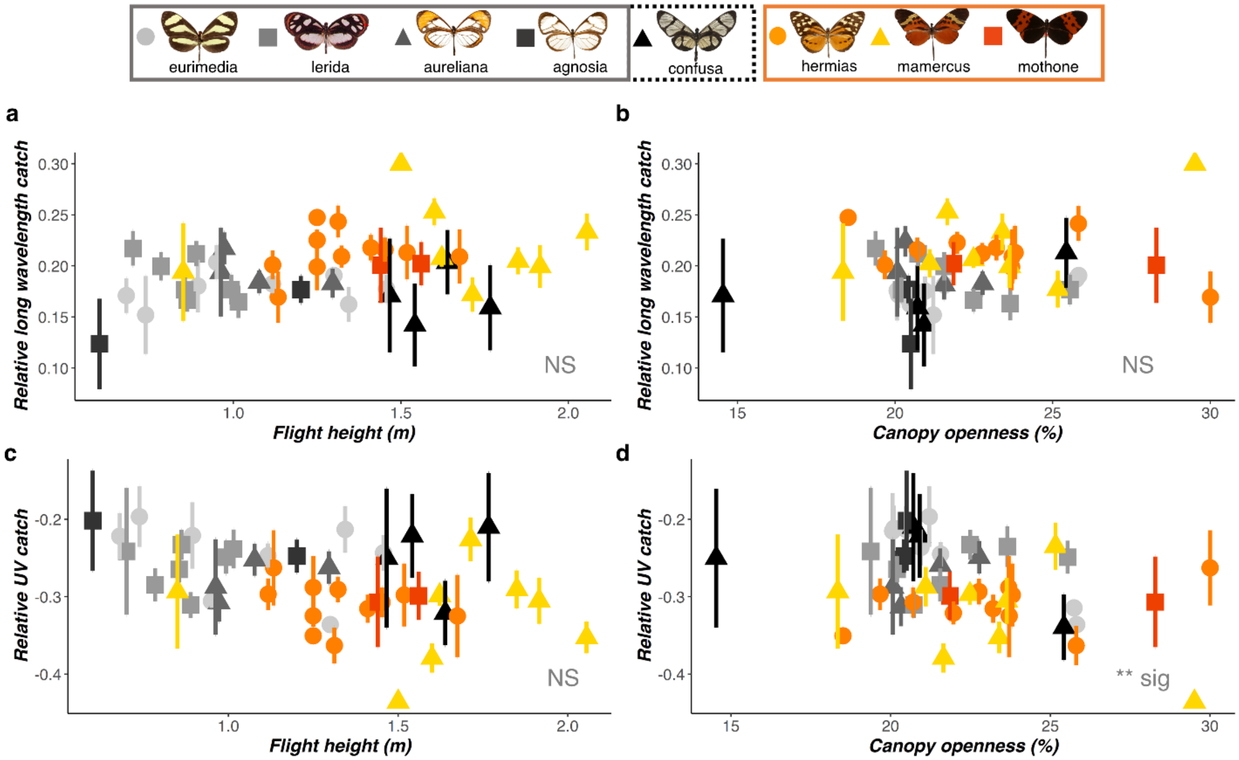

Figure S4. Variation in spectral composition between ithomiine microhabitats. a-d, Mean relative quantum catch of long wavelengths (**a**,**b**) and ultraviolet (**c**,**d**) of each species (*N* = 785, 45 species), coded by mimicry ring, plotted against mean flight height (m; **a**,**c**) and canopy openness (%; **b**,**d**). Error bars indicate standard error. Mimicry rings were shown to significantly segregate with respect to the relative catch of long wavelengths, but not ultraviolet. Example models for each mimicry ring are shown on the top row, grouped based on their general color pattern classification (grey = 'clearwing', dotted black = 'confusa', orange = 'tiger-stripe'). Significance of each ecological variable is indicated at the bottom right of each panel. NS P > 0.05, *P < 0.05, **P < 0.01, ***P < 0.001.

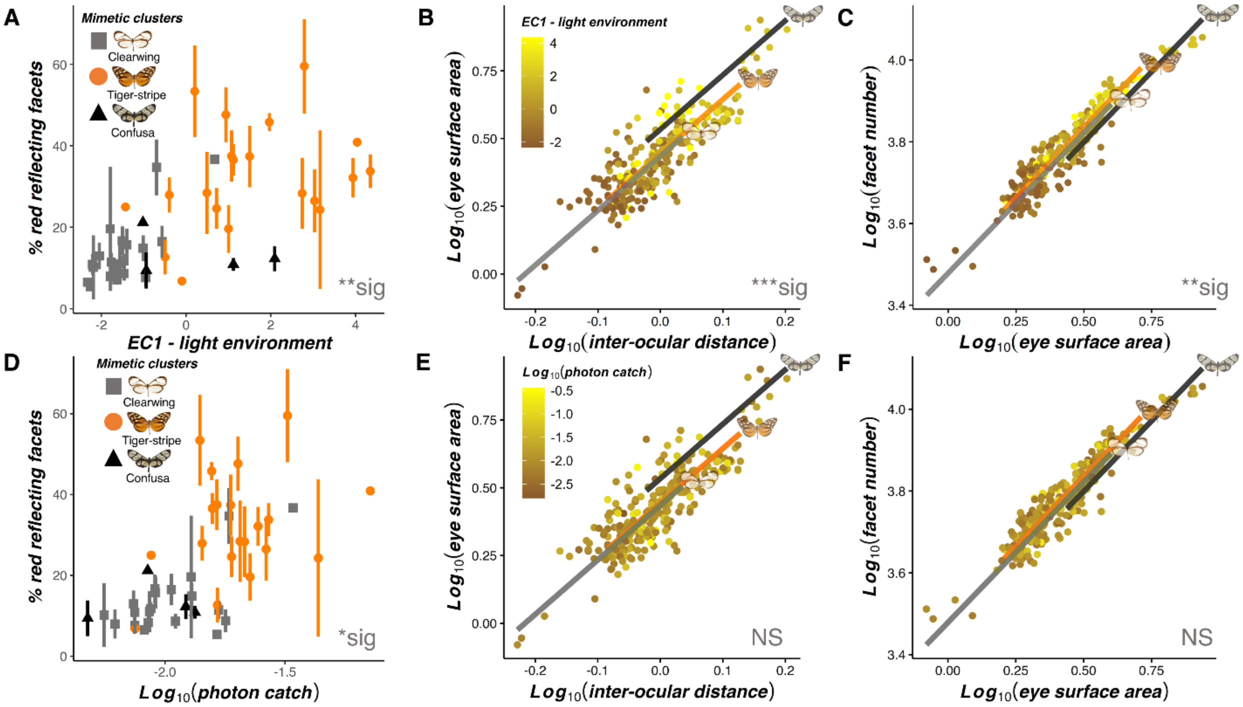

Figure S5. Additional eye physiological and anatomical associations with light environment and mimicry (*N* = 363, 45 species). a-f, Correlation of traits with respect to EC1 for each species (**a-c**) and (log_{10}-transformed) photon catch (10^{10} quanta s^{-1} m^{-2}) for the LW sensitivity function (**d-f**), showing differences in the proportion of red reflecting facets, shown as species means coded by mimetic cluster (grey = 'clearwing', black = 'confusa', orange = 'tiger-stripe') (**a**,**d**), eye surface area (mm^2) when scaled against inter-ocular distance (mm) (**b**,**e**), and number of facets when scaled against eye surface area (**c**,**f**). For each panel containing an allometric control, all variables are log_{10}-transformed and regression lines for each mimetic cluster, estimated from standardized major axis regression are superimposed on top, alongside example models (**b**,**c**,**e**,**f**). The color scale for EC1 and photon catch are shown on the top left of **b** and **e** respectively. Asterisks at the bottom right of each panel indicate the significance level of EC1 (A-C) and photon catch (D-F) at explaining variation in each trait. NS P > 0.05, *P < 0.05, **P < 0.01, ***P < 0.001.

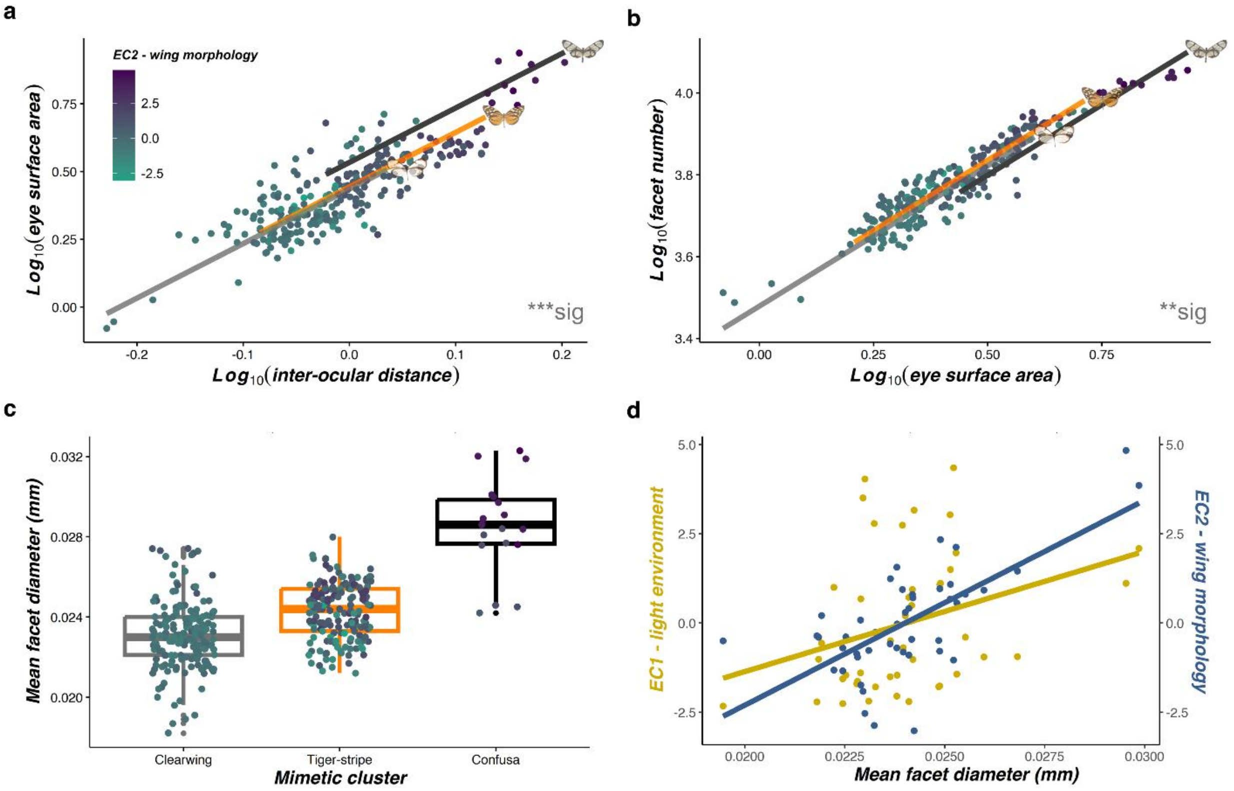

Figure S6. Eye anatomical associations with wing morphology (*N* = 363, 45 species). a,**b**, Non-allometric convergence between EC2 for each species and eye surface area (mm^2), when scaled against inter-ocular distance (mm) (**a**), and facet number, when scaled against eye surface area (**b**). All eye anatomy traits in (**a**,**b**) are log_{10}-transformed. Regression lines for each mimetic cluster (grey = 'clearwing', black = 'confusa', orange = 'tiger-stripe'), estimated from standardized major axis regressions are superimposed on top, alongside example models. **c**, Convergence in mean facet diameter (mm) for individuals with similar wing morphologies, separated by mimetic cluster. Medians (thick horizontal bars), interquartile ranges (boxes), values within 1.5 interquartile ranges of the box edges (whiskers), and possible outliers (datapoints outside whiskers) are plotted. In **a-c**, turquoise-purple color shades represent the mean EC2 value for each species, the color scale for which is shown in the top left of **a**. Asterisks at the bottom right of each panel indicate the significance level of EC2 at explaining variation in each trait. NS $P > 0.05$, $^*P < 0.05$, $^{**}P < 0.01$, $^{***}P < 0.001$. **d**, Positive correlation between the mean facet diameter of each species and both ecological PC axes, representing light environmental (EC1, yellow) and wing morphological variation (EC2, blue).

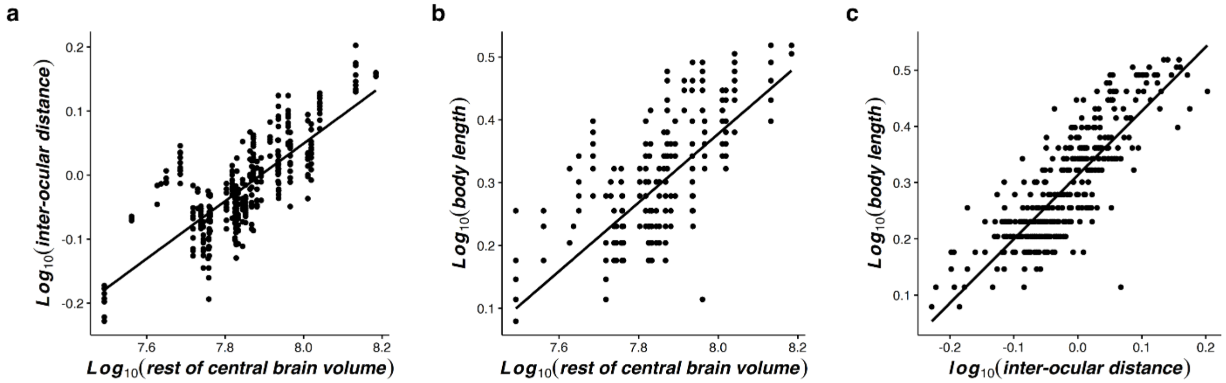

Figure S7. Positive correlations between $\log_{10}$-transformed allometric controls. **a**,**b**, Interocular distance (mm) (**a**) and body length (cm) (**b**) plotted against the mean "rest of central brain" volume (μm^3) of each species. **c**, Body length plotted against inter-ocular distance. All traits are $\log_{10}$-transformed.

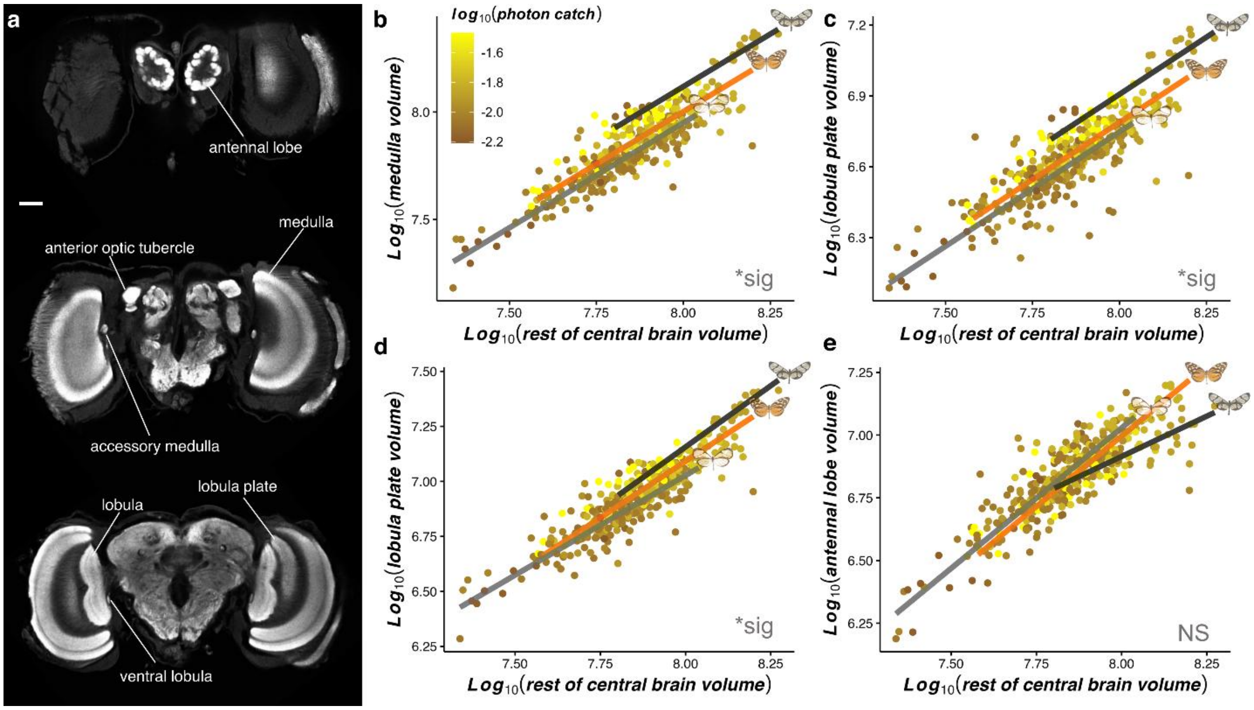

Figure S8. Additional neuroanatomical associations with light environment and mimicry (*N* = 374, 40 species). **a**, Anti-synapsin immunofluorescence from frontal confocal brain sections of *Ithomia amarilla*, with imaging performed progressively along the posterior axis, moving from top to bottom. All reconstructed neuropils are labelled. Scale bar = 100 µm. **b-e**, Nonallometric convergence between the mean overall photon catch (10^{10} quanta s^{-1} m^{-2}) for the LW sensitivity function of each species and the level of volumetric investment (μm^3) in the medulla (**b**), lobula plate (**c**), lobula (**d**), and antennal lobe (**e**) when scaled against the volume of the "rest of central brain". Brown-yellow color shades represent the mean photon catch for each species, the color scale for which is shown in the top left of **b**. All variables are log_{10}transformed. Regression lines for each mimetic cluster (grey = 'clearwing', black = 'confusa', orange = 'tiger-stripe'), estimated from standardized major axis regressions are superimposed on top, alongside example models. Asterisks at the bottom right of each panel indicate the significance level of photon catch at predicting relative investment in each neuropil. NS P > 0.05, *P < 0.05, **P < 0.01, ***P < 0.001.

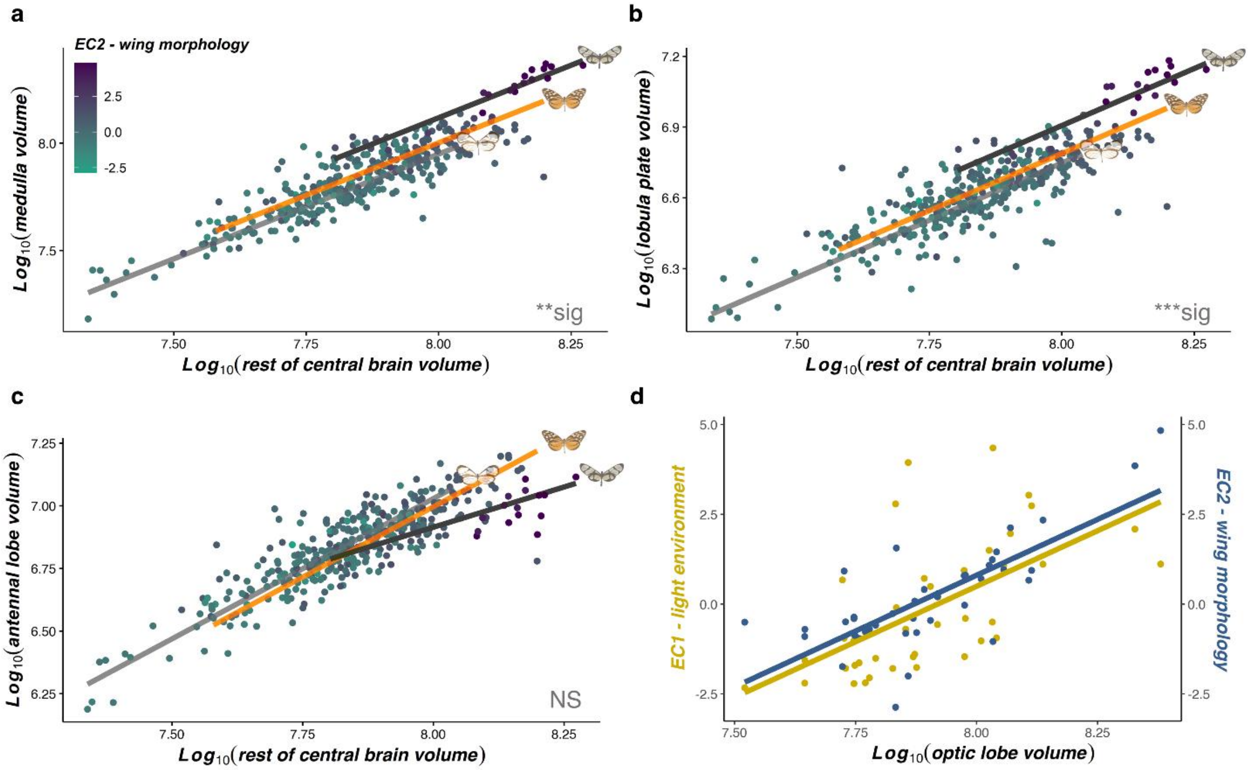

Figure S9. Neuroanatomical associations with wing morphology (*N* = 374, 40 species). a-c, Non-allometric convergence between the mean wing morphology of each species and the volume (μm^3) of the medulla (**a**), lobula plate (**b**), and antennal lobe (**c**), when scaled against the volume of the "rest of central brain". All volumetric traits are $\log_{10}$-transformed. Turquoise-purple color shades represent the mean EC2 value for each species, the color scale for which is shown in the top left of **a**. Regression lines for each mimetic cluster (grey = 'clearwing', black = 'confusa', orange = 'tiger-stripe'), estimated from standardized major axis regressions are superimposed on top, alongside example models. Asterisks at the bottom right of each panel indicate the significance level of EC2 at explaining variation in each neuropil. NS $P > 0.05$, *$P < 0.05$, **$P < 0.01$, ***$P < 0.001$. **d**, Positive correlation between the mean gross optic lobe volume of each species and both ecological PC axes, representing light environmental (EC1, yellow) and wing morphological variation (EC2, blue).

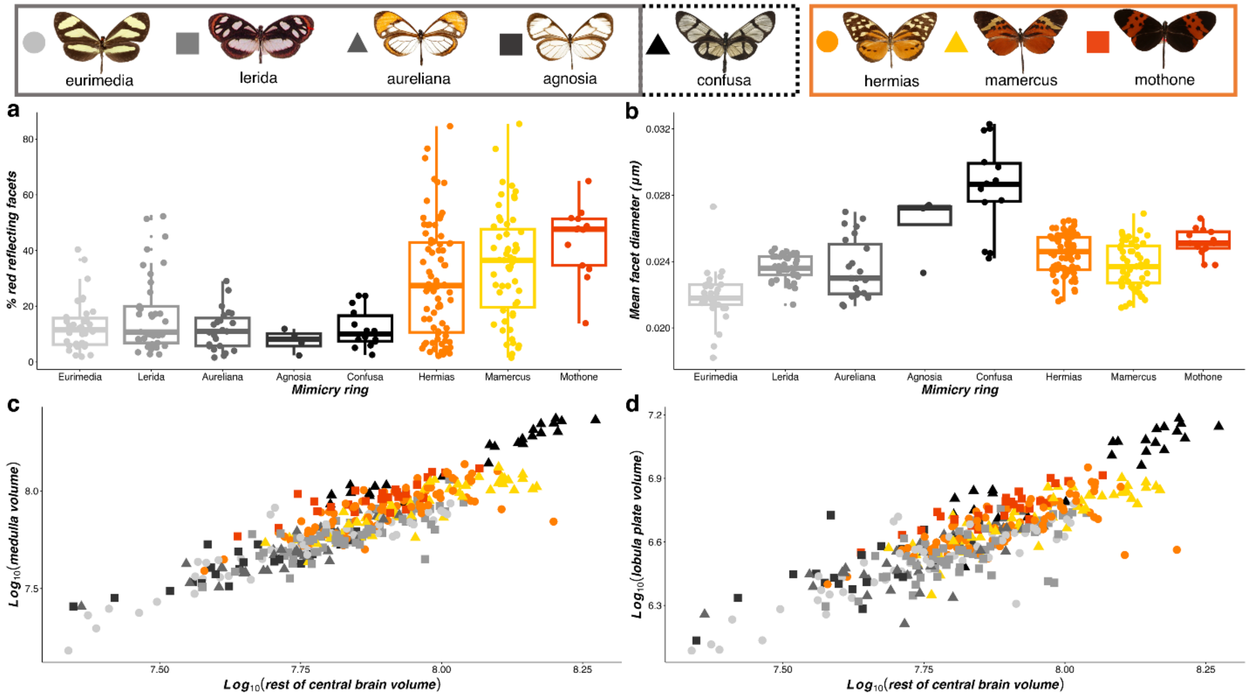

Figure S10. Convergence in visual traits within and between mimicry rings. Example models for each mimicry ring are shown on the top row, grouped based on their mimetic cluster (grey = 'clearwing', dotted black = 'confusa', orange = 'tiger-stripe'). **(a,b)** Convergence in the proportion of red-reflecting facets (%) from eyeshine images and mean facet diameter (µm). Medians (thick horizontal bars), interquartile ranges (boxes), values within 1.5 interquartile ranges of the box edges (whiskers), and possible outliers (datapoints outside whiskers) are plotted. **(c,d)** Non-allometric convergence in the volume (μm^3) of the medulla **(c)** and lobula plate **(d)**, when scaled against the volume of the "rest of central brain". All volumetric traits are log_{10}-transformed.

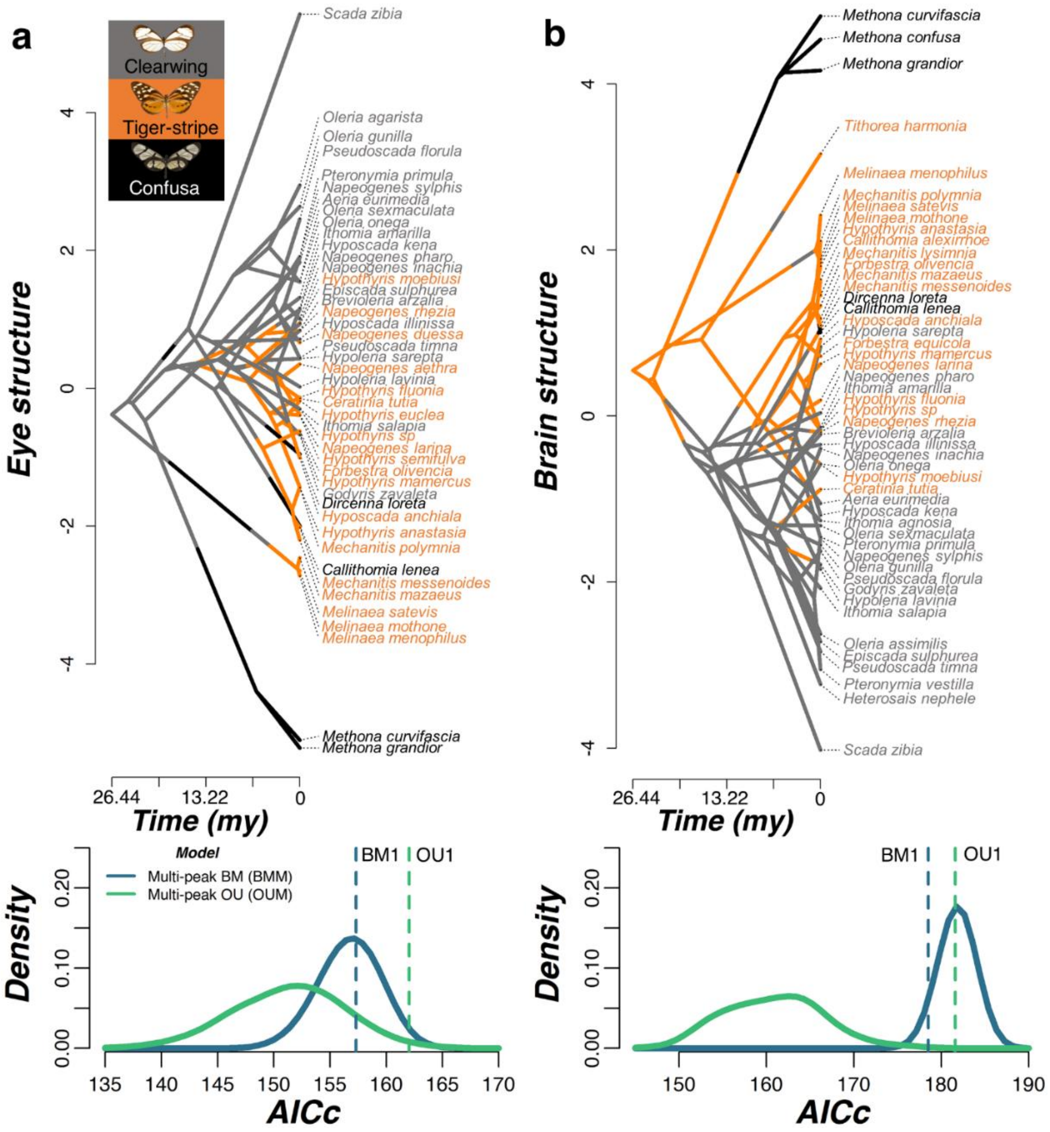

Figure S11. Convergence in eye and brain structure separately among co-mimics. a,**b**, Above are phenograms based on PC1 of two principal component analyses which summarized variation in eye (*N* = 45 species) (**a**) and brain (*N* = 49 species) (**b**) structure based on traits which showed significant light environmental effects. Instances where branches cross and concentrate in a given area indicate convergent lineages. Below each phenogram are Kernel density plots of small sample corrected Akaike Information Criterion (AICc) scores obtained from multi-peak Brownian motion (BM) and Ornstein-Uhlenbeck (OU) evolutionary models, constructed from 500 simulated character maps where species belonging to the same mimetic cluster were assigned to the same selective regime. Blue and green vertical dashed lines indicate the AICc score for single-peak BM (BM1) and OU (OU1) models respectively. Example models of each mimetic cluster are shown in the top left of **a**.

Supplementary dataset appendix

Supplementary Datasets (Dataset SX.xlsx)

Dataset S1. Light microhabitat variation and partitioning along a topographically variable transect. (A) Linear mixed model output showing the effect of canopy openness, height from the ground, and topography (and their interaction) at explaining variation in each spectral variable. **(B)** MCMCglmm output, including pairwise comparisons, showing how the abundance of ithomiine mimicry rings differs with respect to ecological variation along the transect. **(C)** Scores and loadings from a principal component analysis, included in the above analysis, conducted on estimated photon catches of each photoreceptor for spectral measurements taken along the transect.

Dataset S2. Light microhabitat variation and partitioning for individually caught ithomiine butterflies. a) MCMCglmm and PGLS segregation analyses. MCMCglmm (i) and PGLS (ii) output, including pairwise comparisons, showing the effect of canopy openness, flight height, and mimicry ring at explaining visual niche variation for each spectral variable. **b) spectral PCA and light-wing morphological associations.** (i) Scores and loadings from a principal component analysis, included in the above analysis, conducted on estimated photon catches of each photoreceptor for spectral measurements taken for individual butterflies. (ii) MCMCglmm and PGLS output showing the effect of wing morphological variables at explaining visual niche variation for each spectral variable.

Dataset S3. Ecological principal component analysis (PCA). (A) MCMCglmm and **(B)** PGLS output, including pairwise comparisons, showing the effect of canopy openness, flight height, and mimicry ring at explaining variation in each wing morphological variable.

Dataset S4. Visual system associations with light environment and flight-related morphology a) ecological PC axes. MCMCglmm (i) and PGLS (ii) output showing the effect of EC1 (light environment) and EC2 (wing morphology) at explaining variation in each visual trait, alongside allometric controls where appropriate. (iii) MCMCglmm and PGLS output showing correlations between allometric controls. **b) spectral and wing morphological variables.** MCMCglmm (i,ii) and PGLS (iii, iv) output showing the effect of individual spectral variables (i,iii) and individual wing morphological variables (ii,iv) at explaining variation in each visual trait, alongside allometric controls where appropriate.

Dataset S5. Sensory convergence between co-mimics. (A) MCMCglmm (i) and PGLS (ii) output, including pairwise comparisons, showing the effect of mimetic cluster at explaining variation in each visual trait that showed significant light environmental associations, alongside allometric controls where appropriate. **(B)** Output from standardized major axis regression analysis, including pairwise comparisons, testing for a non-allometric effect of mimetic cluster at explaining the scaling relationship of each visual trait, using individual (i) and species mean (ii) data.

Dataset S6. Visual system principal component analyses (PCA). (A-C) Scores and loadings from PCAs summarizing variation in eye+brain structure **(A)**, and eye **(B)** and brain **(C)** structure separately.

Dataset S7. Evolutionary modelling of convergence. (A) Summarized output from single/multi-peak Brownian motion (BM), Ornstein-Uhlenbeck (OU) and early burst (EB) models, created in *mvMORPH*, for EC1, EC2, eye+brain structure, and eye and brain structure separately, with co-mimics assigned to different selective regimes. **(B)** Raw output from each

mvMORPH model simulation (*N* = 500) **(C)** Convergence (C) indices from a *convevol* analysis, testing for convergence in eye+brain structure.

SI References

1. Govardovskii, V., Fyhrquist, N., Reuter, T., Kuzmin, D. & Donner, K. In search of the visual pigment template, *Visual Neuroscience* **17**, 509-528 (2000).
2. Stavenga, D. G. On visual pigment templates and the spectral shape of invertebrate rhodopsins and metarhodopsins, *Journal of Comparative Physiology A* **196**, 869-878 (2010).
3. Wainwright, J. B., Schofield, C., Conway, M., Phillips, D., Martin-Silverstone, E., Brodrick, E. A., Cicconardi, F., How, M. J., Roberts, N. W. & Montgomery, S. H. Multiple axes of visual system diversity in Ithomiini, an ecologically diverse tribe of mimetic, *Journal of Experimental Biology* **226**, jeb246423 (2023).
4. Belušič, G., Ilić, M., Meglič, A. & Pirih, P. Red-green opponency in the long visual fibre photoreceptors of brushfoot butterflies (Nymphalidae), *Proceedings of the Royal Society B-Biological Sciences* **288**, 20211560 (2021).
5. Liénard, M. A., Valencia-Montoya, W. A. & Pierce, N. E. Molecular advances to study the function, evolution and spectral tuning of arthropod visual opsins, *Philosophical Transactions of the Royal Society B Biological Sciences* **377**, 20210279 (2022).
6. Buchsbaum, G. & Gottschalk, A. Trichromacy, opponent colours coding and optimum colour information transmission in the retina, *Proceedings of the Royal Society B Biological Sciences* **220**, 89-113 (1983).

7. Heath, S. L., Christenson, M. P., Oriol, E., Saavedra-Weisenhaus, M., Kohn, J. R. & Behnia, R. Circuit mechanisms underlying chromatic encoding in *Drosophila* photoreceptors, *Current Biology* **30**, 264-275 (2020).
8. Stavenga, D. G. Reflections on colourful ommatidia of butterfly eyes, *Journal of Experimental Biology* **205**, 1077-1085 (2002).
9. Stavenga, D. G., Numan, J. A. J., Tinbergen, J. & Kuiper, J. W. Insect pupil mechanisms. II. Pigment migration in retinula cells of butterflies, *Journal of Comparative Physiology A* **113**, 73-93 (1977).
10. Schindelin, J., Arganda-Carreras, I., Frise, E., Kaynig, V., Longair, M., Pietzsch, T., Preibisch, S., Rueden, C., Saalfeld, S., Schmid, B., Tinevez, J-Y., White, D. J., Hartenstein, V., Eliceiri, K., Tomancak, P. & Cardona, A. Fiji: an open-source platform for biological-image analysis, *Nature Methods* **9**, 676-682 (2012).
11. Ilić, M., Chen, P-J., Pirih, P., Meglič, A., Prevc, J., Yago, M., Belušič, G. & Arikawa, K. Simple and complex, sexually dimorphic retinal mosaic of fritillary butterflies, *Philosophical Transactions of the Royal Society B Biological Sciences* **377**, 20210276 (2022).
12. Montgomery, S. H. & Ott, S. R. Brain composition in *Godyris zavaleta*, a diurnal butterfly, reflects an increased reliance on olfactory information, *Journal of Comparative Neurology* **523**, 869-891 (2015).
13. Revell, L. J. phytools: An R package for phylogenetic comparative biology (and other things), *Methods in Ecology and Evolution* **3**, 217-223 (2012). (doi:10.1111/j.2041-210X.2011.00169.x)
14. Stayton, C. T. Analysis of convergent evolution, R package version 1.3, https://CRAN.R-project.org/package=convevol (2018).
